# Brain Regeneration Resembles Brain Cancer at Its Early Wound Healing Stage and Diverges From Cancer Later at Its Proliferation and Differentiation Stages

**DOI:** 10.3389/fcell.2022.813314

**Published:** 2022-02-10

**Authors:** Yeliz Demirci, Guillaume Heger, Esra Katkat, Irene Papatheodorou, Alvis Brazma, Gunes Ozhan

**Affiliations:** ^1^ Izmir Biomedicine and Genome Center (IBG), Dokuz Eylul University Health Campus, Inciralti-Balcova, Izmir, Turkey; ^2^ Izmir International Biomedicine and Genome Institute (IBG-Izmir), Dokuz Eylul University, Inciralti-Balcova, Izmir, Turkey; ^3^ Wellcome Sanger Institute, Hinxton, United Kingdom; ^4^ École Centrale de Nantes, Nantes, France; ^5^ European Molecular Biology Laboratory–European Bioinformatics Institute (EMBL-EBI), Cambridge, United Kingdom

**Keywords:** wound healing, proliferation, differentiation, zebrafish, low-grade glioma (LGG), glioblastoma, comparative transcriptome analysis

## Abstract

Gliomas are the most frequent type of brain cancers and characterized by continuous proliferation, inflammation, angiogenesis, invasion and dedifferentiation, which are also among the initiator and sustaining factors of brain regeneration during restoration of tissue integrity and function. Thus, brain regeneration and brain cancer should share more molecular mechanisms at early stages of regeneration where cell proliferation dominates. However, the mechanisms could diverge later when the regenerative response terminates, while cancer cells sustain proliferation. To test this hypothesis, we exploited the adult zebrafish that, in contrast to the mammals, can efficiently regenerate the brain in response to injury. By comparing transcriptome profiles of the regenerating zebrafish telencephalon at its three different stages, i.e., 1 day post-lesion (dpl)-early wound healing stage, 3 dpl-early proliferative stage and 14 dpl-differentiation stage, to those of two brain cancers, i.e., low-grade glioma (LGG) and glioblastoma (GBM), we reveal the common and distinct molecular mechanisms of brain regeneration and brain cancer. While the transcriptomes of 1 dpl and 3 dpl harbor unique gene modules and gene expression profiles that are more divergent from the control, the transcriptome of 14 dpl converges to that of the control. Next, by functional analysis of the transcriptomes of brain regeneration stages to LGG and GBM, we reveal the common and distinct molecular pathways in regeneration and cancer. 1 dpl and LGG and GBM resemble with regard to signaling pathways related to metabolism and neurogenesis, while 3 dpl and LGG and GBM share pathways that control cell proliferation and differentiation. On the other hand, 14 dpl and LGG and GBM converge with respect to developmental and morphogenetic processes. Finally, our global comparison of gene expression profiles of three brain regeneration stages, LGG and GBM exhibit that 1 dpl is the most similar stage to LGG and GBM while 14 dpl is the most distant stage to both brain cancers. Therefore, early convergence and later divergence of brain regeneration and brain cancer constitutes a key starting point in comparative understanding of cellular and molecular events between the two phenomena and development of relevant targeted therapies for brain cancers.

## Introduction

Despite decades of research, primary brain tumors are still the most difficult-to-treat and deadliest types of cancer. They can occur due to the continual uncontrolled proliferation of brain cells including neurons and glial cells. About 240,000 cases of brain and nervous system-related cancers are diagnosed worldwide every year (Boffetta et al., 2014). Among these, gliomas, arising from glial tissue, are the most frequently occurring type of tumors in the central nervous system (CNS) and responsible for 80% of all malignant primary brain and CNS cancers (Schwartzbaum et al., 2006; Agnihotri et al., 2013; Ostrom et al., 2013; Boffetta et al., 2014; Messali et al., 2014; Hanif et al., 2017). Gliomas are classified into grade I to IV by WHO according to their histopathological and immunohistochemical similarities to the putative cell of origin. Whereas grade I gliomas are less aggressive and slow-growing, grades II to IV are more aggressive, malignant and invasive (Louis et al., 2016; Hanif et al., 2017; Louis et al., 2021). Grade IV gliomas, also known as glioblastoma (GBM), are the most aggressive diffuse forms of all gliomas and account for more than 50% of adults diagnosed with glioma (Louis et al., 2016; Tamimi and Juweid, 2017; Louis et al., 2021). Genetic and environmental factors including age, gender, ethnicity, inherited susceptibility, immune factors and prior radiation have been associated with the risk of developing glioma (Bondy et al., 2008; Prasad and Haas-Kogan, 2009; Ostrom et al., 2013; Tamimi and Juweid, 2017; Ladomersky et al., 2019). While some types of gliomas such as pilocytic astrocytoma are more prevalent in children and young adults, the incidence of GBM increases with advancing age (Merchant et al., 2010; Das and Kumar, 2017; Jones et al., 2017; Ladomersky et al., 2019). In addition to common mutations in the genes *isocitrate dehydrogenase* (*IDH*) *1* and *2*, and *telomerase reverse transcriptase* (*TERT*), progression of glioma has been associated with alterations in various pathways that are crucial to today’s treatments for glioma/glioblastoma (Idilli et al., 2017): 1) alterations in the PI3K-PTEN-Akt-mTOR signaling pathway regulated by epidermal growth factor (EGF) and its receptor EGFR (Zoncu et al., 2011; Li et al., 2016), 2) mutations in the p53 pathway that promote excessive cell cycle progression and prevent apoptosis (Ohgaki and Kleihues, 2007; Viotti et al., 2014; Speidel, 2015), 3) mutations in *NF1, BRAF, RAF1, MEK, PDGFR* and *RTK* genes that affect RAS/MAPK signaling pathways (Venkatesan et al., 2016; Nasser and Mehdipour, 2018) and 4) changes in the genes regulating cell cycle and cell growth such as *retinoblastoma protein* (*pRB*), *cyclin-dependent kinase 4 (cdk4)* and *cyclin-dependent kinase inhibitor 2A* (*cdkn2A*) (Mao et al., 2012; Idilli et al., 2017; Nasser and Mehdipour, 2018).

Despite the modern therapies, curing brain tumors is still a considerable challenge due to the tumor heterogeneity, presence of blood-brain barrier (BBB) and missing pieces in the underlying molecular mechanisms. Among these tumors, GBM remains one of the deadliest cancer types, having a very poor prognosis with a median survival of about 15 months from the diagnosis and a 5-year survival rate of only 5% in adults (Ohka et al., 2012; Fernandes et al., 2017; Tamimi and Juweid, 2017; Wang et al., 2021). GBM treatment consists of a complex multidisciplinary approach including maximal surgical resection followed by radiation therapy and chemotherapy. After resection, applying radiotherapy together with temozolomide (TMZ) is the most effective combinatorial treatment that has been shown to extend survival (Stupp et al., 2005; Fernandes et al., 2017). Combinations of conventional therapies and new approaches targeting several molecular events, such as triggering of apoptosis and suppression of angiogenesis, can improve the prognosis of patients with GBM (Fernandes et al., 2017; Tamimi and Juweid, 2017). Nevertheless, for over 4 decades, the outcomes of GBM treatment have remained stable, necessitating rapid development of new therapeutic approaches.

*Cancer* and regeneration have been historically linked as both processes are triggered with the same biological phenomenon, i.e. cell proliferation. Historically, cell proliferation had first been proposed as a mechanistic link between development, regeneration and cancer by Waddington in the early 1930s (Waddington 1935; Stern, 2000). Due to the cellular similarities between tumor stroma and granulation tissue, which forms at the wound site, cancers have long been described as wounds that do not heal (Haddow, 1972; Dvorak, 1986; Schafer and Werner, 2008). A proper regeneration process is terminated in a controlled manner so that the regenerating tissue does not transform into a mass of cells that undergo uncontrolled proliferation. If regeneration cannot be processed or terminated properly, the tissue might -as in the case of cancer-undertake continuous proliferation due to chronic injury, hypoxia and inflammation and cannot re-establish tissue integrity (Dvorak, 1986; Coussens and Werb, 2002; Beachy et al., 2004; Gurtner et al., 2008; Schafer and Werner, 2008; Oviedo and Beane, 2009; Verkhratsky and Butt, 2013). In contrast to the limited ability of the human brain to regenerate, the non-mammalian vertebrate zebrafish can regenerate the CNS throughout its life (Grandel et al., 2006; Diotel et al., 2020). This ability of the adult zebrafish brain is maintained by the existence of stem/progenitor cells that can continuously proliferate and a permissive environment for neurogenesis (Kizil et al., 2012b). While mammalian adult neurogenesis is restricted to only two regions of the forebrain, i.e., the subventricular zone (SVZ) of the lateral ventricles in the telencephalon and the subgranular zone (SGZ) of the dentate gyrus in the hippocampus, zebrafish has sixteen distinct proliferative niches located in the ventricular zone and deeper in the brain parenchyma with self-renewing neural progenitors (Kriegstein and Alvarez-Buylla, 2009; Bonfanti and Peretto, 2011; Kaslin et al., 2017; Zambusi and Ninkovic, 2020). Thus, this high regenerative capacity of the zebrafish brain constitutes a unique platform to compare the transcriptome of a healing brain at its different stages with that of continuously growing/metastasizing brain tumors. To address this striking issue, we have first set out to identify the genes that are differentially expressed in the adult zebrafish telencephalon at the following three stages of brain regeneration in response to stab wound injury: the early wound healing stage at 1 day post-lesion (dpl), the early proliferative stage at 3 dpl and the late differentiation stage at 14 dpl. We have identified 6,123, 4,662 and 1954 differentially expressed genes (DEGs) at 1, 3 and 14 dpl, respectively. A vast majority of the DEGs identified at all three stages were upregulated. Using Gene Ontology (GO) term and Kyoto Encyclopedia of Genes and Genomes (KEGG) pathway analyses, we have identified that neurogenesis-related genes were prominent among DEGs at 1 dpl. While 3 dpl was marked by the genes related to immune response, cell proliferation and apoptosis, genes with key roles in neuronal differentiation and the Notch pathway were abundant among DEGs at 14 dpl. Weighted gene co-expression network analysis (WGCNA) of three regeneration stages revealed twelve distinct co-expression modules, nine of which were specific to a particular stage. Moreover, gene modules and gene expression profiles at 1 dpl and 3 dpl were unique, while those at 14 dpl are rather similar to the control group. Next, we have compared the whole transcriptomes of the regenerating brain at the three stages to those of the human adult brain tumors low-grade glioma (LGG) and glioblastoma (GBM). The early wound healing stage was similar to brain cancer with respect to activation of metabolic responses and neurogenesis-related signaling pathways. The early proliferative stage and brain cancers shared DEGs related to cell proliferation. The differentiation stage was similar to cancer with respect to activation of developmental and morphogenetic processes. Finally, our comparative transcriptomics and functional analyses of the genes that are differentially expressed in at least one stage of brain regeneration and shared with at least one type of brain cancer have revealed that the stage that most resembled the brain cancer was the early wound healing stage (1 dpl) and that the similarity decreased at the later stages of brain regeneration. Overall, by revealing the stage-dependent similarities and discrepancies between brain regeneration and brain cancer, our study paves the way to test the potential of specific molecular mechanisms of regeneration to stop cancer.

## Materials and Methods

### Stab Wound Assay and Sample Collection

**FIGURE 1:**
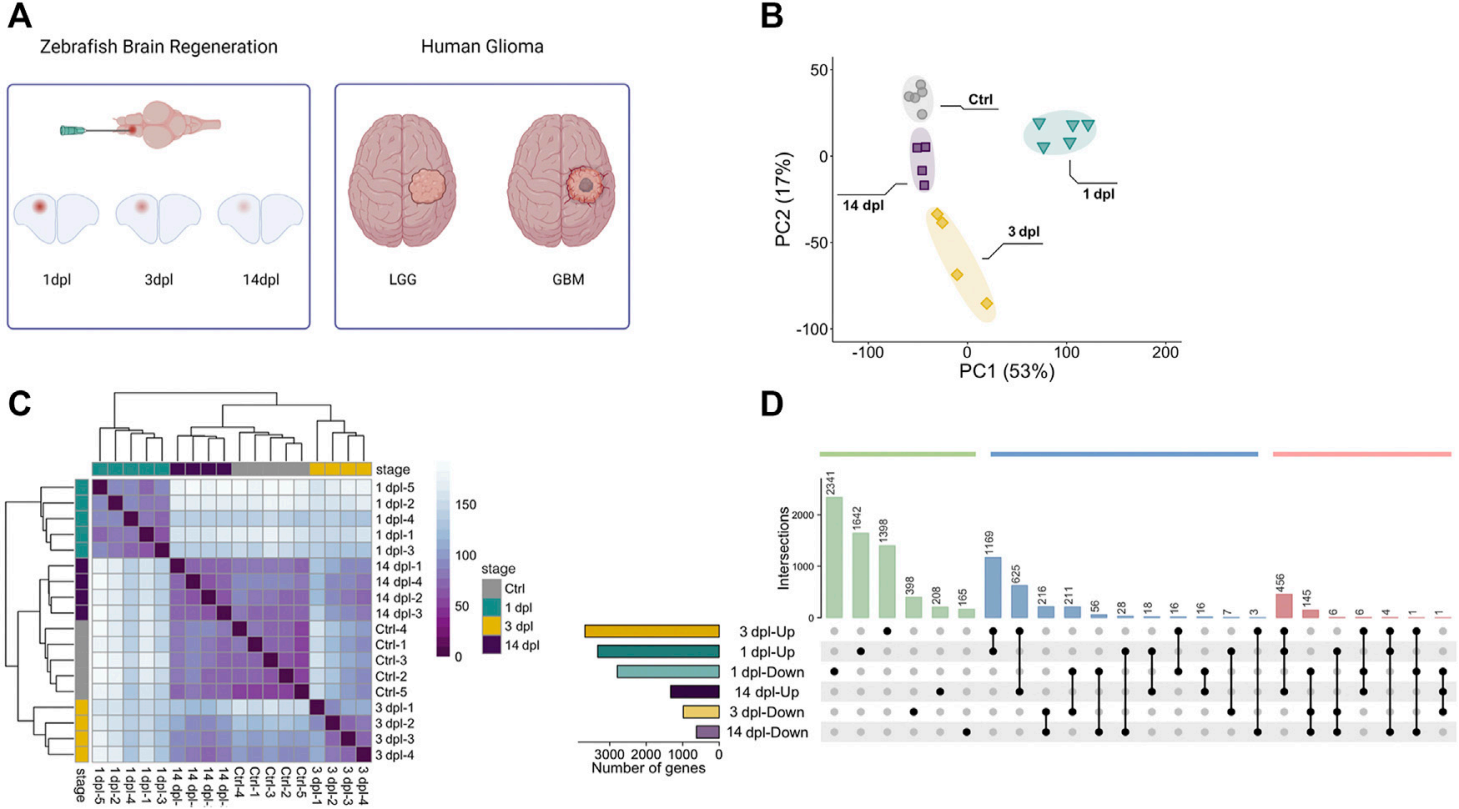
Sample preparation from three different stages of zebrafish brain regeneration and initial analyses of the transcriptome data. **(A)** Generation of the stab lesion and preparation of the RNA samples from lesioned hemispheres at 1, 3 and 14 dpl. Transcriptomes of the regenerating brain were compared to those of human adult LGG and GBM. **(B)** Principal component analysis (PCA) of three brain regeneration stages and their controls. Different colors of circle, square or rectangle dots represent the four groups of samples. Four or five dots with the same color refer to the biological replicates of a sample group. Four sample groups were well clustered among their replicates and well separated from other sample groups. **(C)** Sample-to-sample distance heatmap generated by using normalized counts for overall gene expression patterns for three stages of brain regeneration and control brain samples generated by the DESeq2 package. Different colors of dots represent the four groups of samples. **(D)** UpSet plot shows the comparison of DEG sets between regeneration stages. Total number of DEGs as Up or Down and time points are shown on *x* and *y* axes, respectively. Green bars represent the genes unique to a time point, blue bars the intersection of genes between two different time points and red bars the intersection of genes between three different time points. Black dots connected by lines correspond to the time point and Up/Down state. Numbers of overlapping genes are shown above each bar. dpl: days post-lesion, ctrl: control, LGG: low-grade glioma, GBM: glioblastoma, Up: upregulated, Down: downregulated.

Stab injury was performed in 6–10 month-old wild-type (wt) AB zebrafish as previously described (Kroehne et al., 2011; Baumgart et al., 2012). Before generating a lesion, fish were anaesthetized with 0.02% (w/v) of tricaine methanesulphonate (Supelco, PA, United States) (Schmidt et al., 2014). Stab wound injury was generated by inserting a 30-gauge needle through the left nostril up to the caudal end of the telencephalon (Figure 1A). Following injury, the fish were transferred into a tank of freshwater. At 1, 3 or 14 dpl of stab injury, zebrafish were re-anaesthetized with 0.02% (w/v) of tricaine solution and euthanized by submersion in ice water for 5 min (Schmidt et al., 2014). After extracting the whole telencephalon tissue, lesioned (left) hemispheres were dissected and collected individually in RNAprotect tissue reagent (Qiagen, Germany) to prevent RNA degradation. The left hemispheres of healthy zebrafish telencephalons were used as control samples. All stab lesions were performed on the same day, and fish were sacrificed at corresponding time points from that moment (1, 3 or 14 dpl). Control fish were sacrificed on the day of the stab lesion. Experiments were carried out in quadruplets for each group. Zebrafish were raised and handled in accordance with the guidelines of the Izmir Biomedicine and Genome Center’s Animal Care and Use Committee. Animal experiments were inspected and approved by the Animal Experiments Local Ethics Committee of Izmir Biomedicine and Genome Center (IBG-AELEC).

### RNA Isolation and cDNA Preparation

Following removal of RNAprotect, 700 µL of Qiazol reagent (Qiagen, Germany) was added on the brain tissues and the tissues were homogenized by using a sterile disposable pestle. Total RNA was isolated using the RNeasy Plus Micro Kit (Qiagen, Germany) according to the manufacturer’s instructions and quantified with a NanoDrop 2000 spectrophotometer (Thermo Scientific, MA, United States). RNA integrity and quality was measured by using an Agilent RNA 6000 Pico kit in a 2,100 Bioanalyzer (Agilent Technologies, CA, United States) following the manufacturer’s instructions.

### Library Construction and RNA Sequencing (RNA-Seq)

The samples with an RNA Integrity Number (RIN) ≥ 8 were selected for RNA sequencing (RNA-seq). RNA quality was further tested by performing quantitative reverse transcription PCR (RT-PCR) with a primer pair producing an 812-bp product for zebrafish *beta actin 1* (*actb1*) as a housekeeping gene. To work with equal amounts, RNA samples were adjusted to 100 ng. Samples that passed the quality control steps were sent to the Genomics Core Facility (GeneCore, EMBL Heidelberg, Germany) for library preparation and RNA-seq. Libraries were prepared with an Illumina TruSeq RNA Library Preparation Kit v2 (Illumina, San Diego, CA, United States) according to the manufacturer’s instructions. 500 ng of cDNA was used for each reaction. A paired-end, strand-specific sequencing platform was used on an Illumina NextSeq 500 (Illumina, CA, United States) with a read length of 75 bp.

### Quantitative Polymerase Chain Reaction

To validate the differentially expressed genes obtained via RNA-seq analysis within the original RNA samples, RNA was converted to cDNA by using the ProtoScript II First Strand cDNA Synthesis kit (New England Biolabs, MA, United States). qPCR was performed in triplicates by using GoTaq qPCR Master Mix (Promega, WI, United States) in an Applied Biosystems 7,500 Fast Real-Time PCR machine (Thermo Fisher Scientific, MA, United States). Expression values of each sample were normalized to *Danio rerio ribosomal protein L13a (rpl13a)*. The efficiency of each primer pair was assessed by using the standard curve assay according to the relevant program of the machine. Standard curve with C_T_ values were generated by using the ABI software and a correlation coefficient (*R* ^2^) was calculated for each primer pair. Primer pairs with the *R* ^2^ values equal to or greater than 0.99 and an efficiency falling in the acceptable range (90–110%) were used in the qPCR reactions. Data were analyzed with the GraphPad Prism 8 software (Graphpad Software Inc., CA, United States). The values are indicated as mean ± SEM (Standard Error of Mean) of triplicates. Primer sequences for the tested zebrafish genes are provided in Supplementary Table S1.

### Transcriptomic Analyses of Zebrafish Brain Regeneration and Human Brain Cancers

Read quality control of each zebrafish brain RNA-seq sample was initially performed by using the FastQC tool (Andrews, 2010). The reads were aligned to the zebrafish reference genome GRCz11 (danRer11) using HISAT2 (version 2.1.0) (Kim et al., 2015). After mapping, transcripts were counted with HTSeq 0.6.0 tool by using the annotation file *Danio*_rerio.GRCz11.93. gtf obtained from the Ensembl (Anders et al., 2015). Normalization and transformation (vst) of the read counts, as well as differential expression analysis, were performed by using DESeq2 package (version 1.28.1) of Bioconductor (Love et al., 2014). Principal component analysis (PCA) and sample-to-sample distance analysis were conducted to check data and plots were visualized by using ggplot2 (version 3.3.2) and pheatmap package (version 1.0.12) (Kolde, 2012; Wickham, 2016) (Figures 1B,C). To find differentially expressed genes (DEGs), Wald tests were performed on DESeq2 for the following comparisons: 1) 1 dpl lesioned hemisphere vs. control, 2) 3 dpl lesioned hemisphere vs. control, and 3) 14 dpl lesioned hemisphere vs. control. Secondly, to analyze human brain cancer data, a count matrix was generated using the count data of low-grade glioma (LGG) and glioblastoma (GBM) samples downloaded from The *Cancer* Genome Atlas (TCGA). To identify DEGs, the samples of TCGA-LGG and/or TCGA-GBM projects were compared with the normal tissue samples (control) of the same project. Genes were tested for differential expression using a Wald test with DESeq2 for the following comparisons: 1) TCGA-LGG vs. control and 2) TCGA-GBM vs. control. For all comparisons, genes were marked as upregulated for the fold change >1.5 and downregulated for the fold change <0.67 (= 1/1.5) and for Benjamini–Hochberg adjusted *p*-value (FDR) < 0.05, which will thereafter be referred to as “FC > 1.5 in either directions”.

### Weighted Gene Co-Expression Network Analysis

We ran WGCNA on a filtered and transformed expression matrix of the zebrafish brain regeneration dataset. Raw counts were transformed using the variance-stabilizing transformation (vst) of the R package DESeq2 (Love et al., 2014) as recommended by the WGCNA manual (Langfelder and Horvath, 2008). Genes with less than 10 counts in more than 90% of the samples were filtered for subsequent analysis. After this filtering, 22,853 genes were fed to WGCNA for the regeneration dataset. Network was constructed using unsigned co-expression similarities between genes. As opposed to signed co-expression, unsigned co-expression conserves similarity between highly correlated genes, even in the case of negative correlation. Unsigned co-expression similarity between two genes *i* and *j* is defined as the absolute value of their sample correlation: $s_{i,j}=|cor\left(x_{i},x_{j}\right)|$ . A soft threshold (also called power) of 9 was picked due to the sample size (*n* = 18) accepted as small according in order to construct a co-expression network. The soft threshold β expresses the way the co-expression similarity translates into an adjacency weight in the network: $a_{i,j}=s_{i,j}^{\beta }$ . The higher the soft threshold, the further weak co-expressions are pushed towards 0, although without being made equal to 0, i.e., soft thresholding. For zebrafish brain regeneration data, a power of 9 was chosen by default, due to the sample size (*n* = 18) accepted as small according to the WGCNA manual (Langfelder and Horvath, 2008). A weighted co-expression network was constructed using these parameters. Gene modules were then delineated from the clustering using the dynamic hybrid tree cut algorithm with a deep split parameter of 2 and a minimum cluster size of 100. In other words, modules are defined by pruning the hierarchical clustering dendrogram and grouping the genes that fall in the same branch together. Depending on the parameters, WGCNA merges modules that show similar patterns.

### Collection of Brain Cancer Samples’ Data from The Cancer Genome Atlas

*RNA-seq data of adult human GBM and LGG samples were obtained from the TCGA data portal (* *National Cancer Institute, 2020* *). TCGA defines LGG as tumors of grades II and III based on standards set by the World Health Organization (WHO).* The “Level 3” gene expression data for all TCGA-LGG (529 LGG samples and 4 control samples) and TCGA-GBM (165 GBM samples and 5 control samples) samples were downloaded from the TCGA database.

### Ortholog Conversion

To compare the events measured in the zebrafish and human models, a table of unambiguous orthologous genes was generated between *Homo sapiens* and *Danio rerio* by using BioMart annotations (Smedley et al., 2009). The orthology table obtained from BioMart was first filtered to keep only the pairs of genes indicated with high confidence or with similarities in genes names. The resulting table was further filtered to resolve ambiguities so that each zebrafish gene is assigned a unique human ortholog. For a given zebrafish gene that does not have a human ortholog with the same gene name, a unique human ortholog is selected by ranking the orthology metrics with the following order of priority: gene order conservation score, whole genome alignment coverage, percentage of identity of zebrafish gene to human gene, percentage of identity of human gene to zebrafish gene. Finally, to reduce the number of human genes matched to multiple zebrafish genes, only high confidence pairs were retained (Supplementary Table S7).

### Functional Annotation

**FIGURE 2:**
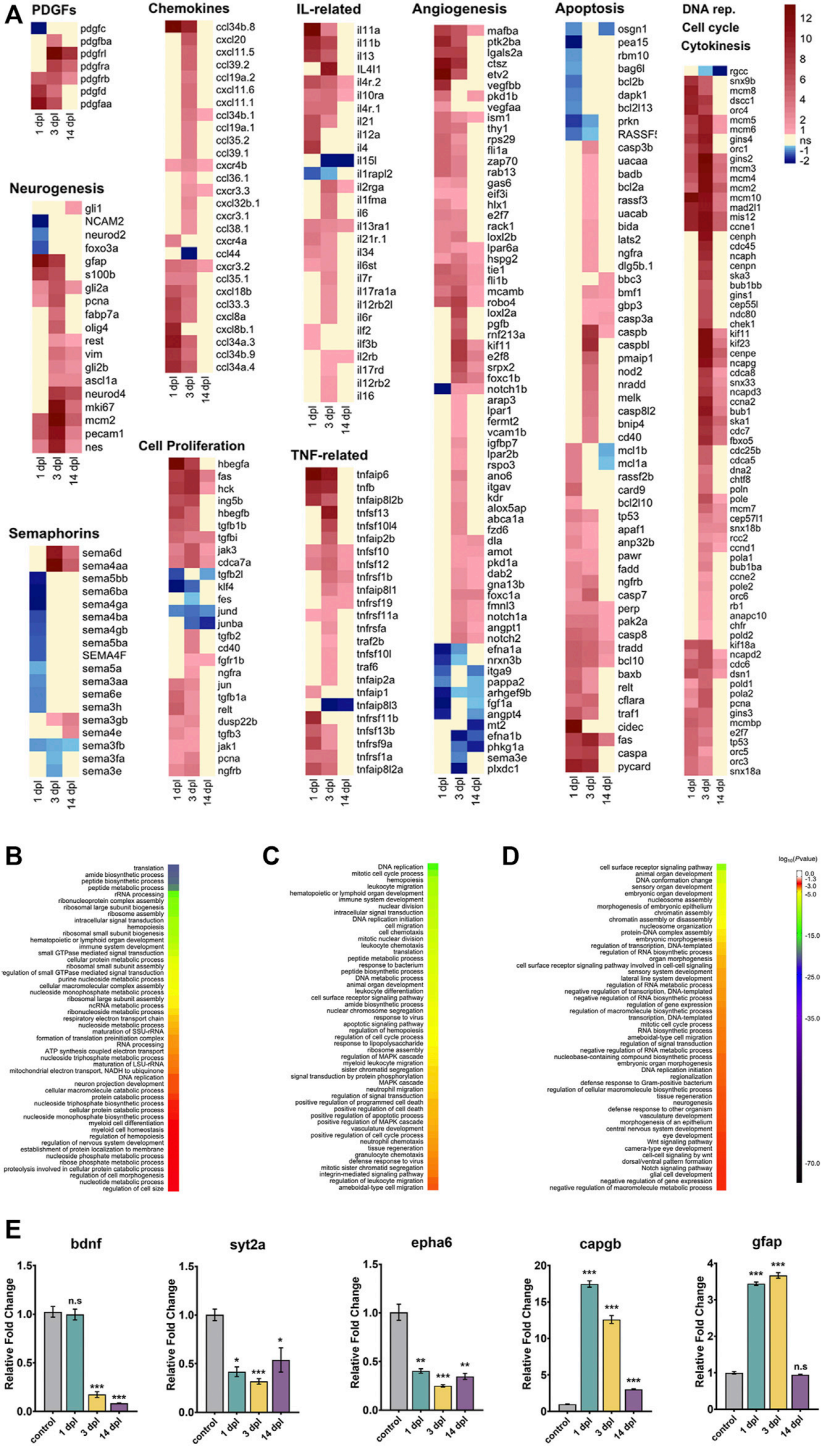
Transcriptome profiling and functional annotation of the telencephalon during early wound healing (1 dpl), early proliferative (3 dpl) and differentiation (14 dpl) stages of zebrafish brain regeneration. **(A)** Heatmaps of log_2_ fold changes of selected genes across three stages of brain regeneration. Each column represents a time point and each row shows a single gene. The scale bar shows red for Up, blue for Down, yellow for weak regulation (FC < 1.5 in either direction) or statistically non-significant (Benjamini–Hochberg adjusted *p*-value (FDR) > 0.1). **(B–D)** GO-BP terms enriched at 1 dpl **(B)**, 3 dpl **(C)** and 14 dpl **(D)** by using all DEGs. DAVID was used to show the most significantly enriched GO-BP terms. All DEGs (1 dpl: 6,123, 3 dpl: 4,662, 14 dpl: 1954) were used for the analyses. The heatmap scale shows log_10_ of the ease *p*-values for the most significantly enriched GO terms. **(E)** Relative expression levels of genes that are Down or Up at different stages of regeneration. *bdnf* is Down at 3 dpl and 14 dpl, while *syt2a* and *epha6* are Down at all stages. *capgb* is Up at all stages, while *gfap* is Up at 1 dpl and 3 dpl. Statistical significance was evaluated using unpaired *t*-test. *p < 0.05, **p < 0.01 and ***p < 0.001. ns: non-significant. Error bars represent ±standard error of mean (SEM, *n* = 3). Up: upregulated, Down: downregulated, dpl: days post-lesion, DAVID: database for Annotation, Visualization and Integrated Discovery, GO: Gene Ontology, BP: Biological Process.

The lists of significantly altered genes acquired from individual comparisons were used as inputs of functional analyses for the database for Annotation, Visualization and Integrated Discovery (DAVID version 6.8) (Huang da et al., 2009). When comparing stages of zebrafish brain regeneration and human cancers, lists of shared or exclusive genes were built using human orthologs of zebrafish genes (Supplementary Table S7). For comparisons within the zebrafish model, the original gene identifiers were used. For functional enrichment, the ease score, a modified one-tailed Fisher’s exact test, was used to determine the enriched Gene Ontology (GO) terms and Kyoto Encyclopedia of Genes and Genomes (KEGG) pathways by means of a user-defined gene list for each annotated DAVID GO term and KEGG pathway. Functional enrichment was performed according to biological domains of GO terms with respect to three aspects: biological process (BP), molecular function (MF), and cellular component (CC). Gene lists obtained from the AmiGO database (Carbon et al., 2009) and manually curated as related to the selected functions (Figure 2A) were plotted using the R package pheatmap (version 1.0.12) (Kolde, 2012). Gene lists related to the selected KEGG pathways were obtained from the KEGG database and plotted using the GOplot package (Walter et al., 2015). Significantly enriched GO terms and KEGG pathways were plotted using the package ggplot2 (Wickham, 2016).

## Results

### Transcriptome Profiling of Brain Regeneration During Early Wound Healing, Proliferation and Differentiation Stages

Brain regeneration has been analyzed at the transcriptional level in the zebrafish traumatic brain injury model at 5 dpl (Gourain et al., 2021). Moreover, we have recently conducted a comparative transcriptomic profiling of the regenerating zebrafish telencephalon at two early stages of regeneration (Demirci et al., 2020). However, there exists no study that compares the gene expression profiles at the early and late stages of regeneration. Thus, we set out to unravel the dynamic alterations in gene expression that occur from the early wound healing stage (1 dpl), through the proliferative stage (3 dpl) to the late differentiation stage (14 dpl) of brain regeneration (Kroehne et al., 2011; Lindsey et al., 2017; Demirci et al., 2020). To this purpose, we dissected the lesioned (left) hemispheres of the injured zebrafish brain at 1, 3 and 14 dpl, and compared with the equivalent hemispheres of the uninjured control brains. PCA showed clear separation of the samples between control and regeneration stages, which clustered in distinct zones of the principal plane of variance (Figure 1B). The sample-to-sample distance heatmap further supported that the samples exactly matched the main ramifications of the hierarchical clustering (Figure 1C). Among the regeneration stages, samples of 14 dpl positioned most closely to the control samples in both analyses, suggesting that the transcriptome of the late differentiation stage converged to that of the control. Next, we performed differential gene expression analysis. We have detected 6,123 genes (3,330 upregulated [Up] and 2,793 downregulated [Down]), 4,662 genes (3,678 Up, 984 Down) and 1954 genes (1,330 Up, 624 Down) that were differentially expressed in response to injury at 1, 3 and 14 dpl, respectively (Supplementary Figures S1A,C). Differential expression at all stages was asymmetrical in favor of Up genes, with 1 dpl having the highest number of DEGs (Supplementary Table S2). 3,983 DEGs (1,642 Up, 2,341 Down) were unique to 1 dpl (Figure 1D). Heatmaps of selected genes undertaking specific roles during regeneration showed that the Down group at 1 dpl consisted of several neurogenesis-related genes such as *neurod2, olig1, notch3, foxo3a*, *amigo1* and a large number of *semaphorin* genes, encoding for a family of secreted and membrane proteins involved in axonal growth (Supplementary Table S2, Figure 2A). Interestingly, several neural stem/progenitor cell markers including *gfap, nes* and *s100b* were Up, as a sign of reactive neurogenesis (Supplementary Table S2, Figure 2A). At 3 dpl, 1796 DEGs (1,398 Up, 398 Down) were unique (Figure 1D) and mostly consisted of genes related to regulation of apoptosis, cell cycle and cell proliferation (Supplementary Table S2, Figure 2A). Genes related to immune response, chemotaxis and angiogenesis as well as markers of neurogenesis such as *gfap*, *s100b*, *fabp7a*, *neurod4*, *olig4* and *gli* were prominently Up at 3 dpl (Supplementary Table S2, Figure 2A). At 14 dpl, the number of unique DEGs decreased dramatically to 373 (208 Up, 165 Down) (Figure 1D), including the Up neuronal differentiation genes *gli1*, *foxd3*, *her4.2*, *otpb*, *fzd1* and *fzd4* (Supplementary Table S2, Figure 2A). Strikingly, several members of Notch signaling including *notch1a, notch1b, notch2, notchl, her15.1, dla, dlb, dlc, dld, jag1a,* and *jag1b*, were Up at 14 dpl while being Down at 1 dpl (Supplementary Table S2)*,* in accordance with the key roles of Notch signaling in regulation of neuronal differentiation (Imayoshi and Kageyama, 2011).

To investigate the function of the DEGs, we performed GO term enrichment analysis for all three regeneration stages (Supplementary Table S3, Figures 2B–D). At 1 dpl, biosynthetic processes, immune system development and regulation of nervous system development were in the top 50 GO-BP terms (Supplementary Table S3, Figure 2B). KEGG pathways at 1 dpl were also enriched mainly in biosynthetic metabolic pathways as well as several signaling pathways such as mTOR and MAPK (Supplementary Table S4, Supplementary Figure S2). At 3 dpl, top 50 GO-BP terms were enriched mainly in cell cycle, activation of immune response and apoptosis (Supplementary Table S3, Figure 2C). KEGG pathways were likewise enriched in cell cycle, apoptosis, cytokine activation, apoptosis-related p53 signaling and immune response-related JAK-STAT pathway (Supplementary Table S4, Supplementary Figure S2). At 14 dpl, most prominent GO-BP terms were related to organ morphogenesis, neurogenesis, CNS development and vasculogenesis as well as Wnt and Notch signaling pathways (Supplementary Table S3, Figure 2D), which were also enriched in the KEGG pathways (Supplementary Table S4, Supplementary Figure S2). To validate differential gene expression, we selected DEGs that are related to neurogenesis and regulated differently at 1, 3 and 14 dpl. *bdnf*, encoding for a neurotrophic factor, was strongly and selectively Down at 3 dpl and 14 dpl, while the synaptic vesicle protein encoding *syt2a* and ephrin receptor gene *epha6* were Down at all three stages (Figure 2E). On the other hand, regeneration-related capgb was Up at all stages, whereas the glial marker gfap was selectively Up at 1 dpl and 3 dpl (Figure 2E). These results were collectively compatible with the RNA-seq results (Supplementary Table S2).

### Gene Co-Expression Network Analysis Reveals Divergence from Control in Early Stages of Brain Regeneration and Convergence to Control at Late Stages

**FIGURE 3:**
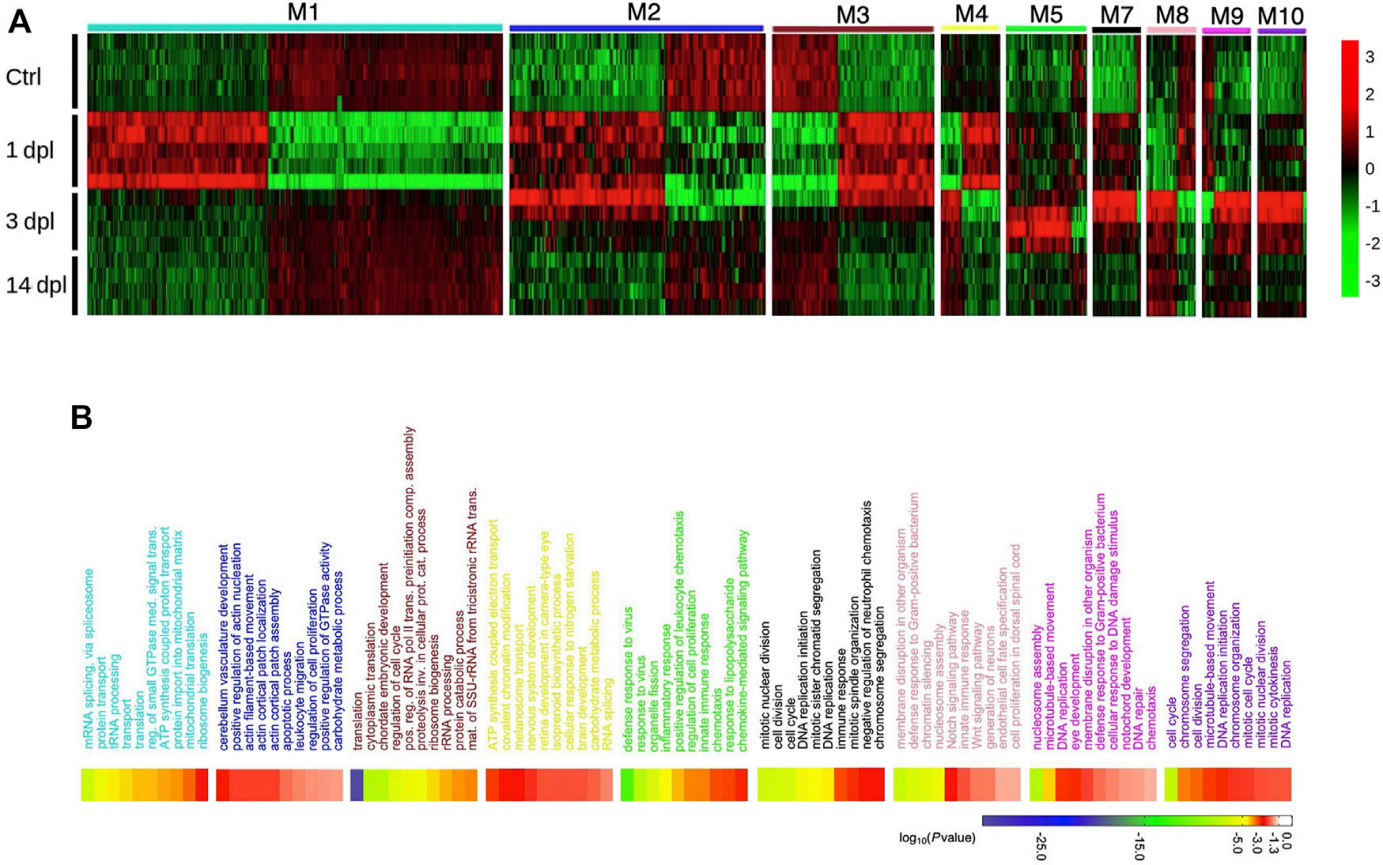
Network analysis of zebrafish brain regeneration at three stages reveals stage-specific modules. **(A)** Heatmap representing relative expression (z-score) of genes that are enriched in each module for the three stages of the adult zebrafish brain regeneration. Each row represents a sample, and each column shows a single gene. Red and green shades show high or low relative expressions, respectively. **(B)** DAVID was used to show the most significantly enriched GO-BP terms based on the transcriptional changes of each significant module and their associated enrichment *p*-values for Top 10 GO-BP terms. The heatmap scale shows log_10_ of the ease *p*-values for the most significantly enriched GO terms. dpl: days post-lesion, ctrl: control.

Next, to explore the co-expression relationship between different gene sets, we performed weighted gene co-expression network analysis (WGCNA) on 1, 3 and 14 dpl samples and identified twelve distinct groups of co-expressed genes, the so-called modules (Supplementary Figure S3, Supplementary Table S5). Expression of the genes clustered in nine modules (M1-M5 and M7-M10) showed a stage-specific component (Figure 3A, Supplementary Figure S3B), i.e., the genes in these modules revealed expression patterns that distinguished one stage of regeneration from the others, indicating a grouped response peaking at that particular stage. Notably, GO term enrichment analyses performed by using the genes clustered in these nine modules showed a similar pattern with that performed by using the DEGs for each stage in BP category (Figure 3B, Supplementary Table S6). For example, genes enriched in M1 (turquoise) and M3 (brown) showed an expression pattern specific to 1 dpl (Figure 3A). GO terms of these two modules were associated with translation and ribosome biogenesis, similar to GO-BP terms obtained from analysis of all DEGs at 1 dpl (compare Figure 3B to Figure 2B). Genes enriched in M5 (green) and M10 (purple) likewise showed a pattern specific to 3 dpl (Figure 3A) and had GO terms enriched in immune response and cell cycle that are compatible with the GO-BP terms generated from all DEGs at 3 dpl (compare Figure 3B to Figure 2C). Genes that were affected at both 1 dpl and 3 dpl were enriched in M2 (blue) and M7 (black) (Figure 3A) and consisted of GO terms related with immune response, cell cycle and apoptosis, which were significantly enriched in GO terms and KEGG pathways performed with genes differentially expressed at one of these stages (compare Figure 3B to Figures 2B,C and Supplementary Figure S2). Interestingly, there was no module specific to 14 dpl, which mostly displayed modules similar to control. Moreover, clustering of regeneration-related GO-BP terms enriched at all three stages further supports that biological events occurring during adult brain regeneration display stage-specific patterns (Supplementary Figure S4).

To understand the changes in gene expression profiles during adult brain regeneration at a global level, we drew heatmap plots using all DEGs (9,136 genes, Supplementary Table S7) identified at three stages by using variance-stabilized counts normalized as z-scores for all samples (Supplementary Figure S5). Control samples showed the lowest variability. Samples of 1 dpl and 3 dpl displayed a high variability, probably due to activation of intense regeneration events such as reactive proliferation, which can vary significantly between individuals. In contrast, the variability decreased in samples of 14 dpl and gene expression patterns became similar to the control, most likely because neuronal circuits are partially re-established at this stage (Kroehne et al., 2011). These data collectively indicate that while 1 dpl and 3 dpl were unique with respect to their gene modules and gene expression profiles, 14 dpl is rather similar to the control group, suggesting that gene expression patterns in later stages of regeneration converge to those of the uninjured state.

### The Early Wound Healing Stage of Brain Regeneration is More Similar to Glioblastoma than to Low-Grade Glioma in Terms of Activation of Metabolic and Neurogenic Pathways

Due to the growing evidence that bridge the mechanisms of regeneration and cancer, we hypothesize that regeneration and cancer must share some molecular mechanisms at the early stages of regeneration where proliferation is the prominent event. However, the mechanisms must diverge later when the regenerative response terminates precisely, while cancer cells keep proliferating. To test whether this hypothesis holds true for the brain, we set out to compare the transcriptome of the regenerating adult brain to that of the brain with cancer. As a first step, we compared LGG/GBM samples from TCGA with normal tissue to identify the DEGs. Expression of 7,992 genes (4,036 Up, 3,956 Down) and 15,469 genes (8,451 Up, 7,018 Down) were significantly altered in LGG and GBM, respectively (Supplementary Figures S6A,B, Supplementary Table S2).

**FIGURE 4:**
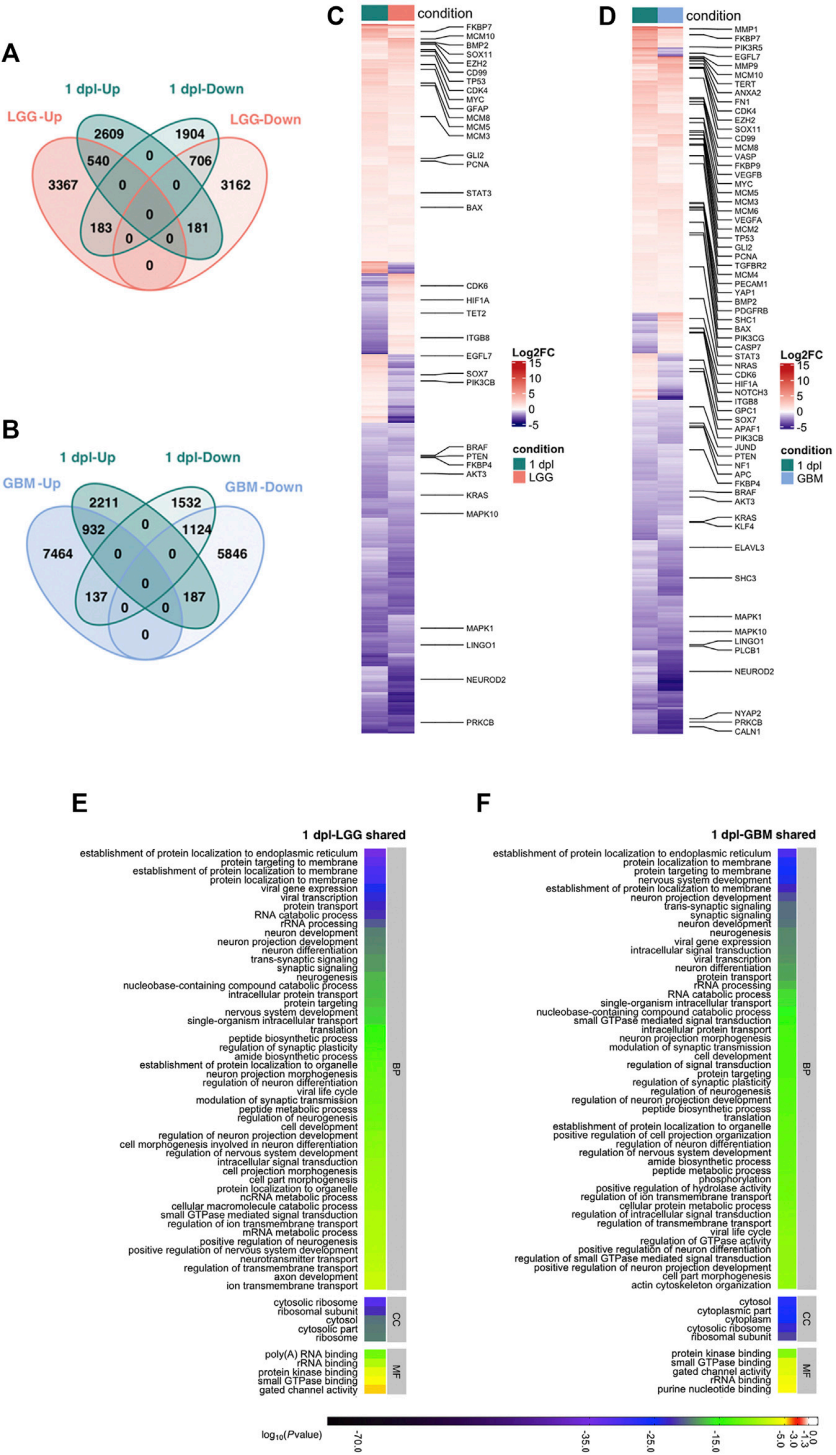
Early wound healing stage (1 dpl) of brain regeneration is similar to brain cancer with respect to induction of metabolism- and neurogenesis-related signaling responses. **(A, B)** Venn diagrams showing the number of upregulated (Up) or downregulated (Down) DEGs and the overlap between 1 dpl (turquoise) and **(A)** LGG (pink) and **(B)** GBM (blue). **(C, D)** Heatmaps show the expression of genes shared between 1 dpl (turquoise) and **(C)** LGG (pink) and **(D)** GBM (blue). Each column represents a condition (1 dpl, LGG or GBM) and each row shows a single gene. The scale bar shows log_2_ fold changes from high to low regulation, represented by a color gradient from red to purple, respectively. **(E, F)** DAVID was used to show the most significantly enriched GO-BP (top 50), CC (top 5), and MF (top 5) terms based on transcriptional changes in comparison of 1 dpl with **(E)** LGG and **(F)** GBM by using human identifiers of shared DEGs. The heatmap scale shows log_10_ of the ease *p*-values for the most significantly enriched GO terms. dpl: days post-lesion, DAVID: database for Annotation, Visualization and Integrated Discovery, GO: Gene Ontology, BP: Biological Process, MF: Molecular Function, CC: Cellular Component, dpl: days post lesion, LGG: low-grade glioma, GBM: glioblastoma.

To investigate the shared genes between early wound healing stage of brain regeneration with LGG and GBM, we intersected unique human orthologs of DEGs at 1 dpl with the DEGs in LGG and GBM. Out of the 6,123 genes that were differentially expressed at 1 dpl, 1,610 genes were shared with LGG and 1,246 of them were altered in the same direction, i.e., both Up or both Down (Figure 4A, Supplementary Table S7). Among shared genes, *tp53*, *gfap*, and *pcna* were Up, while *neurod2, braf, kras, pten* and *akt3* were Down (Figure 4C, Supplementary Table S7). Interestingly, 4,513 genes (2,609 Up, 1904 Down) were unique to 1 dpl. Between 1 dpl and GBM, the number of shared genes increased to 2,380, 2056 of which were regulated in the same direction and included majority of the genes shared between 1 dpl and LGG (Figures 4B,D; Supplementary Table S7). Here, 3,743 genes (2,211 Up, 1,532 Down) were unique to 1 dpl (Figure 4B). Thus, early wound healing stage of regeneration is more similar to GBM than to LGG at the transcriptional level, most likely due to the high number and variation of DEGs detected in GBM.

Next, we performed functional annotation of shared genes by using human gene identifiers (Figures 4E,F, Supplementary Figure S7A; Supplementary Tables S7–S9). 39 terms were shared between top 50 GO-BP terms enriched in the comparisons of shared DEGs in 1 dpl-LGG and 1 dpl-GBM (Figures 4E,F, Supplementary Table S8). These terms included various processes related to protein metabolism and neurogenesis. Furthermore, KEGG pathway enrichment of shared DEGs showed that various neurogenesis-related pathways including mTOR, ErbB, MAPK and oxytocin signaling as well several synapse and axonal pathways were shared between 1 dpl and LGG (Supplementary Figure S7A, Supplementary Table S9). Strikingly, glioma was enriched in shared DEGs of 1 dpl with both LGG and GBM (Supplementary Figure S7A, Supplementary Table S9). To identify KEGG pathways that were specific to the very early stage of brain regeneration, we exploited the DEGs unique to 1 dpl with respect to LGG or GBM. Among the unique top 30 KEGG pathways, apoptosis and the JAK-STAT signaling pathway were prominent (Supplementary Figure S7B, Supplementary Table S10). In summary, our results indicate that the early wound healing stage of brain regeneration is similar to brain cancer with respect to induction of metabolism- and neurogenesis-related signaling responses and different from cancer mainly via induction of apoptosis during early regeneration.

### The Early Proliferative Stage of Brain Regeneration is Similar to Low-Grade Glioma/Glioblastoma with Respect to Active Proliferation

**FIGURE 5:**
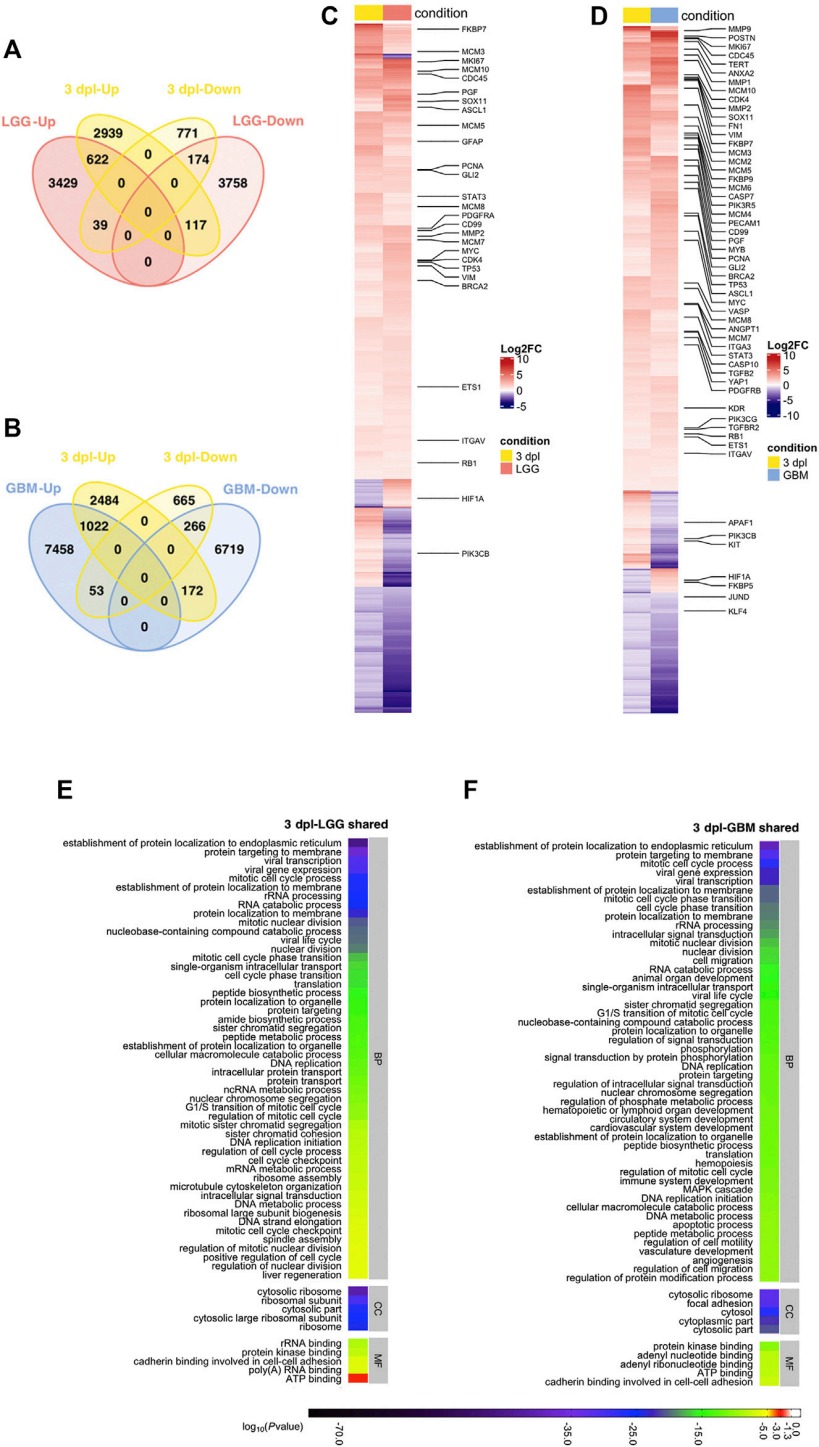
Early proliferative stage (3 dpl) of brain regeneration resembles brain cancer with regard to activation of cell proliferation. **(A,B)** Venn diagrams showing the number of upregulated (Up) or downregulated (Down) DEGs and the overlap between 3 dpl (yellow) and **(A)** LGG (pink) and **(B)** GBM (blue). **(C,D)** Heatmaps showing the expression of genes shared between 3 dpl (yellow) and **(C)** LGG (pink) and **(D)** GBM (blue). Each column represents a condition (3 dpl, LGG or GBM) and each row shows a single gene. The scale bar shows log_2_ fold changes from high to low regulation, represented by a color gradient from red to purple, respectively. **(E,F)** DAVID was used to show the most significantly enriched GO-BP (top 50), CC (top 5), and MF (top 5) terms based on transcriptional changes in comparison of 3 dpl with **(E)** LGG and **(F)** GBM by using human identifiers of shared DEGs. The heatmap scale shows log_10_ of the ease *p*-values for the most significantly enriched GO terms. dpl: days post-lesion, DAVID: database for Annotation, Visualization and Integrated Discovery, GO: Gene Ontology, BP: Biological Process, MF: Molecular Function, CC: Cellular Component, dpl: days post lesion, LGG: low-grade glioma, GBM: glioblastoma.

Next, to reveal the shared genes between the early proliferative stage of brain regeneration and brain cancer, we overlapped human orthologs of DEGs at 3 dpl with the DEGs in LGG and GBM. 952 out of 4,662 DEGs determined at 3 dpl were shared with LGG and 796 out of 952 were Up/Down in both 3 dpl and LGG (Figure 5A, Supplementary Table S7). Shared genes involved the proliferation and glial markers *mki67, pcna*, several *mcm* genes and *gfap*, which were all Up (Figure 5C, Supplementary Table S7). The percentage of unique genes at 3 dpl were greater than that at 1 dpl and reached a total number of 3,710 (2,939 Up, 771 Down) (Figure 5A). When compared to GBM, 1,513 DEGs were shared with 3 dpl and 1,288 of them were altered in the same direction (Figure 5B, Supplementary Table S7). Among the shared Up genes were many proliferative and cancer-related genes such as *angpt1, vim, brca2*, *pcna, mcm2,* and *mki67* (Figure 5D). Here, we found 3,149 DEGs (2,484 Up, 665 Down) that were unique to 3 dpl (Figure 5B).

Functional annotations of shared DEGs revealed that 31 terms out of the top 50 GO-BP terms were mutual between 3 dpl-LGG and 3 dpl-GBM (Figures 5E,F, Supplementary Figure S7A, Supplementary Tables S8, S9). The mutual GO-BP terms contained a number of proliferation-related ones such as various mitotic cell cycle processes, nuclear division processes and DNA replication. GO terms were supported by the KEGG pathway enrichment analysis, which showed that shared DEGs were enriched in various pathways related to proliferation and DNA repair as well as p53, MAPK and calcium signaling pathways (Figure 7B, Supplementary Table S9). Here, several cancer-related pathways were enriched in shared DEGs of 3 dpl with both LGG and GBM (Figure 7A, Supplementary Table S9). Next, we determined the KEGG pathways that are specific to the early proliferative stage of brain regeneration and found that DEGs unique to 3 dpl were enriched in immune response-related processes and apoptosis, p53, Toll-like receptor and JAK-STAT signaling pathways, within the top 30 KEGG pathways (Supplementary Figure S7B, Supplementary Table S10). These data suggest that the early proliferative stage of brain regeneration resembles brain cancer mainly by promotion of cell proliferation, while differing from cancer by the active immune response and apoptosis.

### Developmental and Morphogenetic Signaling Pathways are Commonly Activated During the Differentiation Stage of Brain Regeneration and Low-Grade Glioma/Glioblastoma

**FIGURE 6:**
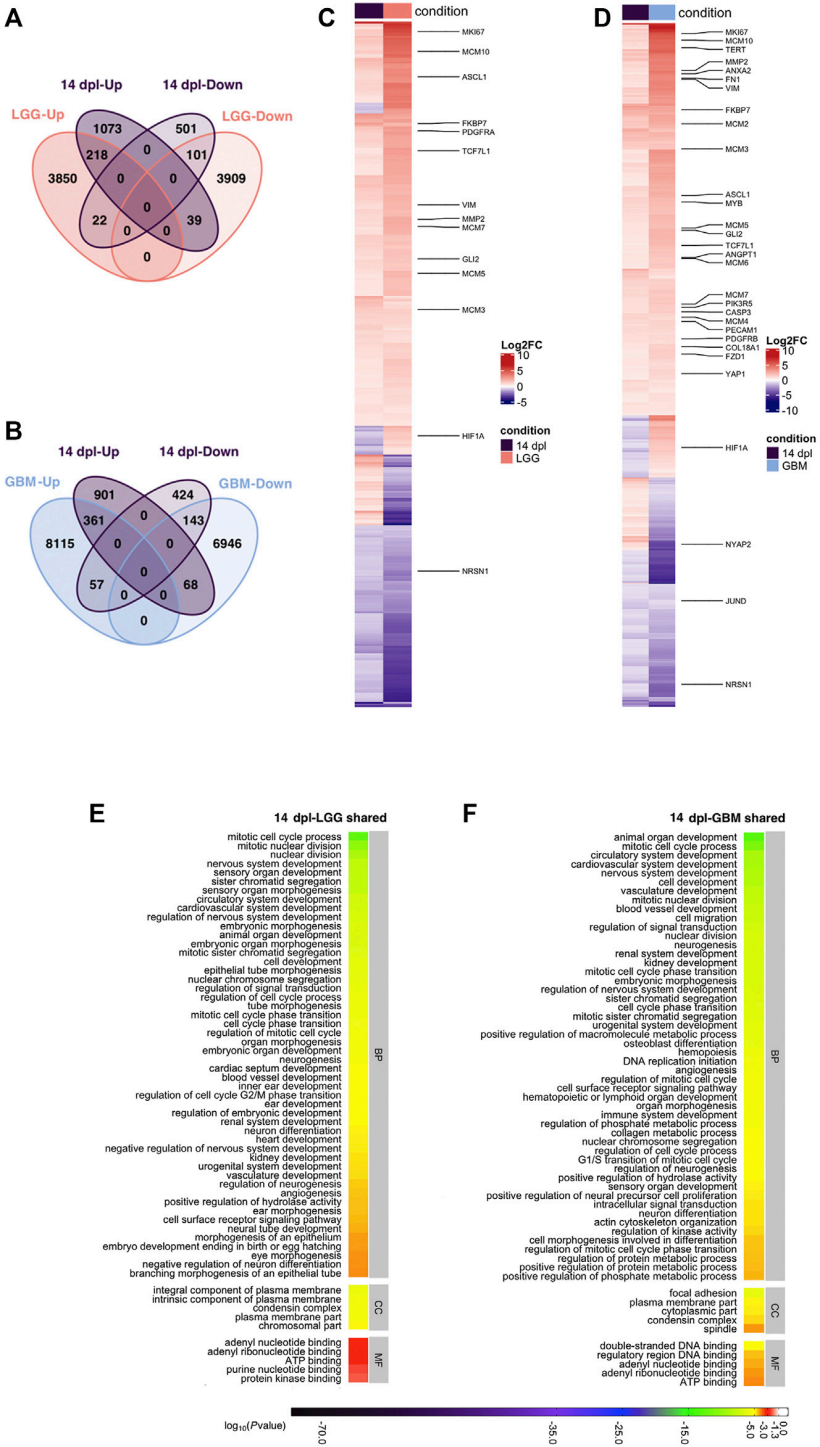
Differentiation stage (14 dpl) of brain regeneration and brain cancer share mechanisms related to developmental and morphogenetic processes. **(A,B)** Venn diagrams showing the number of upregulated (Up) or downregulated (Down) DEGs and the overlap between 14 dpl (purple) and **(A)** LGG (pink) and **(B)** GBM (blue). **(C,D)** Heatmaps showing the expression of genes shared between 14 dpl (purple), and **(C)** LGG (pink), and **(D)** GBM (blue). Each column represents a condition (14 dpl, LGG or GBM) and each row shows a single gene. The scale bar shows their log_2_ fold changes from high to low regulation, represented by a color gradient from red to purple, respectively. **(E,F)** DAVID was used to show the most significantly enriched GO-BP (top 50), CC (top 5), and MF (top 5) terms based on transcriptional changes in comparison of 14 dpl with **(E)** LGG and **(F)** GBM by using human identifiers of shared DEGs. The heatmap scale shows log_10_ of the ease *p*-values for the most significantly enriched GO terms. dpl: days post-lesion, DAVID: database for Annotation, Visualization and Integrated Discovery, GO: Gene Ontology, BP: Biological Process, MF: Molecular Function, CC: Cellular Component, dpl: days post lesion, LGG: low-grade glioma, GBM: glioblastoma.

Next, to compare the differentiation stage of adult brain regeneration with brain cancer, we intersected human orthologs of DEGs at 14 dpl with the DEGs in LGG and GBM. Among 1954 DEGs detected at 14 dpl, 380 were shared with LGG and 319 of the shared DEGs were regulated similarly at 14 dpl and LGG (Figure 6A, Supplementary Table S7). Shared DEGs contained several *mcm* genes and differentiation-related genes (Figure 6C, Supplementary Table S7). 1,574 genes (1,073 Up, 501 Down) were unique to 14 dpl (Figure 6A). 14 dpl and GBM shared 629 genes, 504 of which were regulated in the same direction and mostly overlapped with those shared between 14 dpl and LGG (Figures 6B,D; Supplementary Table S7). 1,325 genes (901 Up, 424 Down) were unique to 14 dpl when compared to GBM (Figure 6B).

Our functional annotation of genes shared between the differentiation stage of brain regeneration and brain cancer demonstrated that 31 of the top 50 GO-BP terms were shared between 14 dpl-LGG and 14 dpl-GBM (Figures 6E,F, S7A, Supplementary Tables S8, S9). A number of GO-BP terms related to development and morphogenesis including nervous system development, neuron differentiation and angiogenesis were remarkable. Moreover, Notch, Wnt, Hippo and calcium signaling pathways were enriched in the top 30 KEGG pathways (Supplementary Figure S7A). Interestingly, several cancer-related pathways were enriched in DEGs between 14 dpl and LGG/GBM (Supplementary Figure S7A, Supplementary Table S9). The Wnt signaling pathway was also enriched in the KEGG pathways that are unique to 14 dpl with respect to LGG/GBM along with p53 and Toll-like receptor signaling (Supplementary Figure S7B, Supplementary Table S10). Thus, signaling pathways that control certain developmental and morphogenetic processes are commonly activated during the differentiation stage of brain regeneration and brain cancer.

### The Early Wound Healing Stage of Brain Regeneration is More Similar to Low-Grade Glioma and Glioblastoma than the Proliferation and Differentiation Stages

**FIGURE 7:**
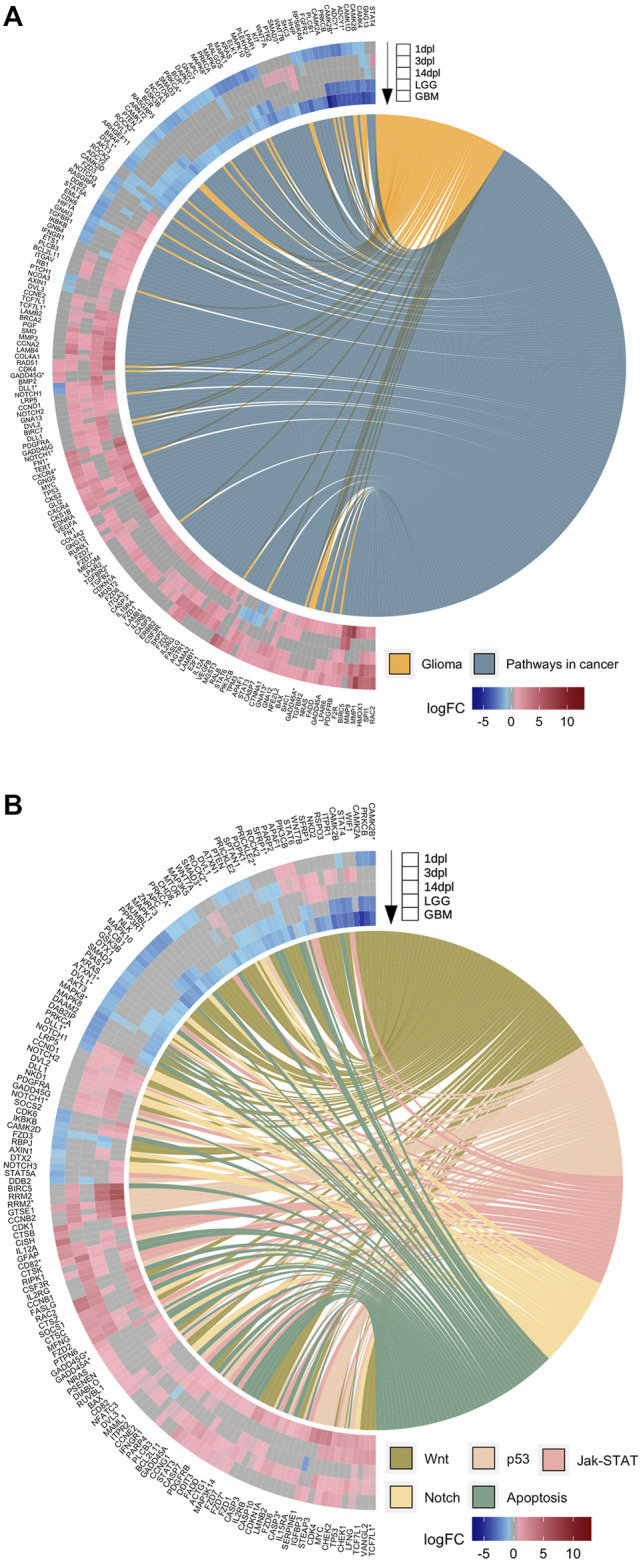
Early wound healing stage of brain regeneration is more similar to LGG and GBM than the proliferation and differentiation stages. **(A,B)** GOChord plots show log_2_ fold changes of the genes annotated in selected KEGG pathways **(A)** “Glioma” and “Pathways in cancer” and **(B)** “Wnt”, “p53”, “Jak-STAT”, “Notch” and “Apoptosis” for three stages of the zebrafish brain regeneration and two types of human brain cancers. The genes are linked to their assigned pathways by ribbons and ordered according to their log_2_ fold change values from high to low regulation, represented by a color gradient from blue to red, respectively. log_2_ fold changes are shown from the outer to the inner annulus in the following order: 1, 3, 14 dpl, LGG and GBM. An asterisk was appended to human genes associated as orthologs to several zebrafish genes in the list. dpl: days post-lesion, LGG: low-grade glioma, GBM: glioblastoma.

While individual comparisons of the regenerative stages to LGG and GBM are informative about particular similarities of these stages to gliomas, a global comparison is necessary to reveal which stage of brain regeneration is most comparable to brain cancer. To this purpose, we drew heatmaps of log_2_ fold changes of the 3,615 genes that are differentially expressed in at least one stage of brain regeneration and shared with at least one type of brain cancer (Supplementary Figure S8). The genes obtained from the KEGG database included a substantial number of genes involved in glioma, pathways in cancer, as well as Wnt, p53, JAK-STAT Notch, apoptosis, RAS, MAPK, mTOR and PI3K-Akt signaling pathways (Figures 7A,B, Supplementary Figure S9). To compare the changes in gene expression associated with these selected pathways in three regenerative stages and two brain cancers, we intersected the genes annotated in these pathways with the DEG sets. Strikingly, the majority of the DEGs of the early wound healing stage showed an expression pattern that is similar to the both human brain cancers, but mostly to GBM (Figures 7A,B, Supplementary Figure S9). In general, if a gene is Up at 1 dpl, it is generally Up in LGG/GBM and if a gene is Down at 1 dpl, it is likewise Down in LGG/GBM (Supplementary Figure S8). The number of significantly altered genes was highest at 1 dpl and decreased at 3 dpl and 14 dpl for all pathways. While most Wnt signaling-related genes were Down or absent across DEG sets, p53 signaling-related genes were mainly Up or absent across DEG sets (Figure 7B). Interestingly, the expression of several genes in the KEGG pathway “pathways in cancer”, such as *ptk2, kit, lpar1, notch1, rasgrp4, ifngr1, ptch1, apaf1* and *dll1*, and Wnt pathway-related genes, such as *wif1, rspo3, nkd2, sfrp1, smad3, wnt7a, wnt7b* and *axin1*, showed opposite expression patterns between brain regeneration and brain cancers, suggesting that these genes may play key roles in preventing the cells from undergoing carcinogenesis (Figures 7A,B). In conclusion, among the three stages of brain regeneration, the early wound healing stage was the most similar one to the brain cancers LGG and GBM with respect to their transcriptomes, while the similarity decreased as regeneration proceeded to the proliferation and differentiation stages.

## Discussion

**FIGURE 8:**
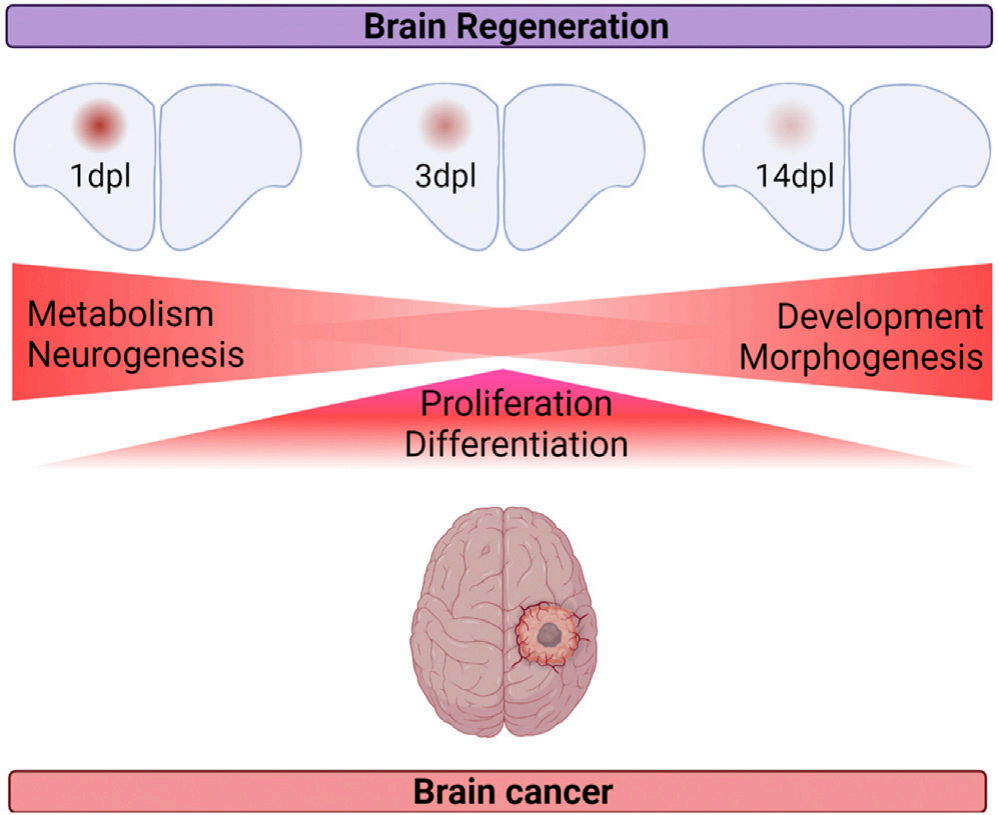
Summary of the shared cellular mechanisms between brain regeneration at three different stages of regeneration and brain cancer.

Despite the studies investigating the common and distinct molecular mechanisms underlying regeneration and cancer, how brain regeneration and brain cancer compare with each other at the level of gene expression has been overlooked. This study has two novel aspects. First, it unravels the gene expression profiles of the regenerating adult zebrafish telencephalon at two early (1 dpl and 3 dpl) and one relatively late (14 dpl) stage of regeneration: 1 dpl as the early wound healing stage, 3 dpl as the early proliferative stage and 14 dpl as the differentiation stage. Second, this study is the first that compares gene expression profiles of the three different stages of adult brain regeneration with two different brain cancers: low-grade glioma (LGG) and glioblastoma (GBM). Based on our detailed analyses, we have drawn the following conclusions: 1) the total number of DEGs at 1 dpl are higher than those at 3 dpl and 14 dpl. 65, 38.5 and 19% of the total DEGs are unique to 1, 3 and 14 dpl, respectively. 2) The more distinctive expression pattern of 1 dpl, and to a lesser extent 3 dpl, is further supported by the unique gene modules that are detected within the transcriptomes of 1 dpl and 3 dpl and by the gene expression profiles that are more divergent from the control. In contrast, the transcriptome of 14 dpl is rather similar to the control group and converges to the transcriptome of the uninjured brain. 3) 1 dpl of brain regeneration is similar to LGG/GBM with respect to activation of metabolism- and neurogenesis-related signaling pathways and different from cancer in the way of activating apoptosis (Figure 8). 4) 3 dpl and LGG/GBM are similar with regard to elevated cell proliferation and differentiation (Figure 8). 5) 14 dpl resembles LGG/GBM because of induced developmental and morphogenetic processes (Figure 8). 6) 1 dpl is more similar to LGG/GBM than 3 dpl and 14 dpl are. Thus, brain regeneration and brain cancer appear to share higher number of molecular mechanisms in the early stages of regeneration, while the similarity decreases at its later stages.

### The Immune Response is Induced Early After Injury and Starts to Decline After the Proliferative Stage

Tissue damage triggers a cascade of early regenerative processes including initiation of wound closure and activation of immune response that is necessary for clearance of tissue debris and deposition of extracellular matrix (Marques et al., 2019). Because of bleeding and inflammation, the lesion site is infiltrated by platelets and immune cells, which are controlled by numerous signaling molecules (Krafts, 2010; Kroehne et al., 2011; Marques et al., 2019). For example, a variety of cells including fibroblasts, macrophages and monocytes, which are primed by mesenchymal stem/stromal cells, are essential for regeneration and activated by the platelet-derived growth factors (PDGFs) to enhance proliferation, chemotaxis and gene expression (Pierce et al., 1991; Andrae et al., 2008; Krafts, 2010). PDGFs have also been shown to be important for myelin regeneration in CNS by stimulating proliferation, differentiation and survival of the cells in the oligodendroglial lineage (Webster, 1997; Watzlawik et al., 2013). Our data showed significant upregulation of PDGF and PDGF receptor (PDGFR) genes such as *pdgfba* selectively at 3 dpl, *pdgfd* and *pdgfaa* at 1 dpl and 3 dpl, and *pdgfrl*, *pdgfra* and *pdgfrb* at 3 dpl and 14 dpl. In addition to the growth factors, cytokines secreted by cells of the immune system act as immunomodulators to regulate the acute inflammatory response that is necessary for functional regeneration of the zebrafish CNS after injury (Krafts, 2010; Kyritsis et al., 2012; Elsaeidi et al., 2014; Fuller-Carter et al., 2015; Tsarouchas et al., 2018). We found several anti-inflammatory cytokines and their receptors including *il6st, il11a, il11b, il13, il21, il21r.1* and *il34* to be significantly upregulated at the two early stages (1 dpl and 3 dpl) of brain regeneration. Moreover, the signature cytokines, including *il12rb2*, *il7r*, *ifng1* and *stat4* (3 dpl) and *il13* and *irf1b* (1 dpl and 3 dpl), for T helper1 (Th1) cell subset are upregulated at the early stages of regeneration (Hamalainen et al., 2001; Duhen et al., 2014; Raphael et al., 2015). Th2 signature cytokines such as *il4* (1 dpl) and *il13* (1 dpl and 3 dpl) were likewise upregulated at the early stages and *ifngr1l* was downregulated at 14 dpl. These findings suggest that both Th1- and Th2-mediated immune responses are activated mainly at the early stages of brain regeneration. Moreover chemokines, a specific type of cytokines, and their receptors play key roles in the activation and infiltration of the immune cells to the injury site in CNS (Jaerve and Muller, 2012). Chemokines have been shown to control immune and progenitor cell homeostasis and thereby regeneration in several zebrafish tissues (Kizil et al., 2012a; Xu et al., 2014; Bussmann and Raz, 2015; Iribarne, 2021). Notably, a number of chemokine and chemokine receptor genes including *cxcl20, cxcl11.5, ccl39.2, cxcl11.6, ccl19a.1* and *ccl36.1* were upregulated at 3 dpl and almost vanished at 14 dpl of brain regeneration. Another group of signaling molecules consists of the members of the tumor necrosis factor superfamily (TNFSF) that are expressed mainly by the immune cells and act as cytokines to regulate neuroinflammation and autoimmunity in the CNS (Sonar and Lal, 2015; Fresegna et al., 2020). Several TNFSF and its corresponding TNFSF receptor superfamily (TNFRSF) genes, such as *tnfb, tnfsf10, tnfsf12, tnfsf13b*, *tnfrsf9a* and *tnfrsf1a*, were significantly upregulated during early regeneration, especially at 3 dpl. The number of altered TNFSF and TNFRSF genes reduced dramatically at 14 dpl. Overall, the parallel activation of PDGFs, cytokines, chemokines and TNF-related factors at the early wound healing stage, their peaking at the proliferative stage and their depletion at the differentiation stage suggest that the immune response is induced early after injury, remains strongly active during establishment of a proliferative response in regeneration and dampens as tissue differentiation starts.

### Activation of Apoptosis Is Regulated in Parallel to Proliferation

Apoptosis is another prominent event that is activated in the early phases of brain regeneration for effective wound healing (Wilson et al., 2007; Guerin et al., 2021). Apoptosis has been shown to be activated twice during early regeneration processes in different organisms. For example, *Hydra* and Planaria appear to have the first peak of apoptosis very early after bisection and the second peak at 3 days after the injury (Chera et al., 2009; Pellettieri et al., 2010; Beane et al., 2013). The adult zebrafish fin regeneration follows a similar route in activation of apoptosis at 12 h post-amputation (hpa) and 72 hpa (Gauron et al., 2013). However, in the *Xenopus* tail regeneration, apoptosis is absent during wound healing, activated at 12 hpa and remains active until 48 hpa (Tseng et al., 2007). We noted a significant upregulation of the apoptosis-related genes *tp53, apaf1, caspa, casp7* and *baxb* at both early regenerative stages, 1 dpl and 3 dpl. Strikingly, the number of apoptosis-related genes doubled at 3 dpl. Apoptosis is considered to have a critical role in resolving inflammation by converting the immune response in early stages of tissue repair into a wound healing response (Brown et al., 1997; Wu and Chen, 2014). Besides, multiple studies have proposed that apoptosis can stimulate proliferation within the regenerating tissues of *Hydra*, Planaria, *Xenopus* and zebrafish (Gargioli and Slack, 2004; Jopling et al., 2010; Morata et al., 2011; Diwanji and Bergmann, 2018; Kha et al., 2018; Stocum, 2019; Guerin et al., 2021). Mainly at 3 dpl, we observed strong activation of apoptosis-related gene expression with a concomitant elevation of cell proliferation. Thus, the capability of the zebrafish telencephalon to convert an early inflammatory reaction into a healing capacity could be reinforced by the parallel elevation in expression level of genes associated with apoptosis at the early wound healing and proliferation stages (Demirci et al., 2020).

### Angiogenic Activity and Proliferation During Brain Regeneration

Angiogenic sprouting into the wound site has been revealed as another essential event of the regeneration process and observed 15 h after injury during heart regeneration in zebrafish (Marin-Juez et al., 2016). *Vascular endothelial growth factor Aa* (*vegfaa*), which is actively involved in angiogenesis, vasculogenesis and endothelial cell growth, is upregulated during heart regeneration of zebrafish (Marin-Juez et al., 2016). Our results revealed upregulation of *vegfaa* specifically at 1 dpl, suggesting that injury triggers a rapid angiogenic sprouting at early brain regeneration. While angiogenesis was strongly promoted at 1 dpl, a massive rise in the number of angiogenesis-related genes was detected at 3 dpl. *Angiopoietin-1* (*angpt1*) has been shown essential to mouse vasculature during response to injury (Jeansson et al., 2011). We found that *angpt1* was upregulated at 3 dpl and 14 dpl. Angiogenesis has been demonstrated to be activated within 4–7 days after cerebral ischemia and contribute to neuronal remodeling and functional recovery via first providing guidance to the sprouting axons through VEGF signaling and second enhancing proliferation, migration and differentiation of neural stem/progenitor cells (Wang et al., 2007; Ruan et al., 2015; Kanazawa et al., 2017; Hatakeyama et al., 2020). Thus, early activation and continued maintenance of angiogenesis during brain regeneration imply a similar role for angiogenesis in the repair of traumatic brain injury.

Adult zebrafish brain regeneration is achieved by injury-induced proliferation of the radial glial cells (RGCs) that gives rise to new neurons (Ghosh and Hui, 2016). RGCs express the glial fibrillary acidic protein (Gfap), an intermediate filament marker of the mammalian astrocytes (Jurisch-Yaksi et al., 2020). Moreover, proliferating cell nuclear antigen (Pcna), a cell proliferation marker, is released by actively dividing RGCs as an indicator of constitutive neurogenesis (Zacchetti et al., 2003). We identified a remarkable increase in the expression of *gfap* and *pcna* during both early stages of regeneration. Besides, *s100b* and *fabp7a*, enriched in quiescent RGC genes, as well as *mki67* (only at 1 dpl) and *mcm2*, markers of dividing cells, were upregulated at the early stages of brain regeneration (Zhang and Jiao, 2015; Kaslin et al., 2017; Lange et al., 2020).

### Brain Regeneration Resembles Brain Cancer at its Earlier Stages and Diverges from Cancer with Regard to Opposite Regulation of Key Cancer-Related Genes

There is growing evidence that associates regeneration with cancer. For example, melanomas have been demonstrated to express genes that have important functions in development of the melanocyte lineage and regeneration of the melanocytes, strongly suggesting that human cancers share features with both development and tissue regeneration (White and Zon, 2008). A previous study in zebrafish has likewise revealed that 40% of the genes that were upregulated during blastema formation in regeneration of the caudal fin are also overexpressed in human melanoma (Hagedorn et al., 2016). However, the underlying mechanistic connection between regeneration and cancer has not been analyzed so far at the molecular level as regard to comparative analysis of the transcriptomes of regenerating brain and brain cancer. The comparison of the three stages of brain regeneration (1, 3 and 14 dpl) with two different brain cancers (LGG and GBM) showed that the number of shared and unique DEGs were the highest in the comparison of 1 dpl with GBM. This is most likely a consequence of the total DEG numbers being highest at 1 dpl and in GBM. Furthermore, the global comparison of the three regeneration stages with two cancers revealed that 1 dpl was the most similar regenerative stage to both LGG and GBM. The DEGs shared between 1 dpl and LGG/GBM were enriched in the KEGG pathway “glioma”. The majority of the genes in this pathway were regulated in the same direction (both Up or both Down) at 1 dpl and LGG/GBM. For example, *Camk2* genes have been found to be strongly downregulated in GBM compared to the normal brain tissue (Johansson et al., 2005; Xiong et al., 2019; He and Li, 2021). *Shc3* and *kras* are likewise downregulated in primary cultures and patient samples of GBM, while *shc1*, *gadd45a* and *tgfbr2* are strongly upregulated (Magrassi et al., 2005; Lymbouridou et al., 2009; Guo et al., 2018; Hirakata et al., 2021). Moreover, the tumor suppressors *pten* and *tp53* are frequently mutated and non-functional in GBM (Benitez et al., 2017; Zhang et al., 2018). Strikingly, the expression of those genes did not change significantly at 3 dpl, nor at 14 dpl. This means that while these genes are essential for the early initiation of a regenerative response upon injury, they need to be suppressed later for the regeneration to be terminated precisely and prevent the transformation of a normal cell into a cancer cell. Thus, the fact that expression of glioma-related genes is similarly regulated exclusively in the early stages of regeneration but not in later stages mark them as drug-targetable candidates for GBM treatment.

Among the shared genes between brain regeneration and brain cancer, a wide range of genes that are related with apoptosis, proliferation, angiogenesis and invasion and have been associated with glioma showed opposite directions of expression regulation. For example, the transcription factor SRY-related HMG-box 7 (Sox7), which acts as a tumor suppressor, has been found to be downregulated in a variety of cancers including GBM and its downregulation has been associated with poor prognosis (Katoh, 2002; Stovall et al., 2013; Liu et al., 2014; Zhao et al., 2016; Oh et al., 2017; Kim et al., 2018). Similarly, apoptosis protease-activating factor-1 (Apaf1), a key molecule in the apoptotic pathways, is downregulated in different cancer types (Soengas et al., 2006; Tanase et al., 2015). In accordance with these findings, we observed downregulation of both *sox7* and *apaf1* in LGG/GBM. However, they were both upregulated at 1 dpl and *apaf1* also at 3 dpl of brain regeneration. In contrast, Hypoxia inducible factor 1 (HIF-1), a key regulator of hypoxia, has been demonstrated to promote the migratory and invasive behavior of glioma cells as well as to induce angiogenesis by regulating the expression of VEGF, PDGFs and PDGFRs (Mendez et al., 2010; Peng et al., 2021). The cell cycle regulator cyclin-dependent kinase 6 (Cdk6) is also known to be significantly upregulated in glioma cells, and its elevated expression correlates with the grades of glioma malignancy and glioma resistance to chemotherapy (Lu et al., 2018). While expression of both *hif-1* and *cdk6* increased in both LGG and GBM, we found them to have decreased in at least one stage of brain regeneration. A recent study showed that overexpression of Annexin A2 (Anxa2) increased the expression of Glypican 1 (Gpc1) via c-Myc, creating a positive feedback loop that enhances proliferation of glioma cells (Li et al., 2021). *Anxa2* expression increased during early regeneration and GBM. Interestingly, while being upregulated in GBM, *gpc1* expression was strongly downregulated at 1 dpl, proposing that the feedback loop activated by Gpc1 in cancer cannot be activated during regeneration. Altogether, these findings strongly suggest that while early brain regeneration is more similar to brain cancer than late regeneration, it also diverges from cancer due to important differences with regard to opposite regulation of key genes related to cancer progression and activation of signaling mechanisms that prevent carcinogenesis.

Glioblastoma stem-like cells (GSCs) are a highly tumorigenic cell group in GBMs and mediate cancer progression, resistance to traditional treatment and recurrence of glioma (Hemmati et al., 2003; Singh et al., 2004; Bao et al., 2006; Gilbert and Ross, 2009; Zhou et al., 2009; Chen et al., 2012). The sustainability of GSCs and progression of glioma rely on the gene that encodes for the Enhancer of Zeste 2 Polycomb Repressive Complex 2 Subunit (EZH2) (Suvà et al., 2009). The transcription factor Signal Transducer and Activator of Transcription 3 (STAT3) is also a key player for propagation and sustainability of multipotency in GSCs (Rahaman et al., 2002; Sherry et al., 2009). EZH2-STAT3 interaction has been shown in GSCs by knockdown of EZH2 using shRNA that causes reduced expression of STAT3 by decreasing H3K27 trimethylation (Kim et al., 2013). EZH2 is also necessary for proliferation of progenitor cells in hippocampal and cortical neurogenesis in mice (Pereira et al., 2010; Zhang et al., 2014). We found that *ezh2* and *stat3* were remarkably elevated during early brain regeneration and LGG/GBM. This suggests that the stem cell characteristics are maintained during early regeneration until cues that direct differentiation are received later.

Semaphorins act as guidance cues during axonal development, and control proliferation, migration and differentiation of neurons during nervous system during development as well as maintenance and function of neuronal circuitries in adult neurogenesis (Carulli et al., 2021). A wide spectrum of roles have been defined for various Semaphorin molecules from regenerative reinnervation to the control of adult neuronal plasticity. For example, Sema3g is necessary for establishment of neural circuit stability and cognitive functions (Tan et al., 2019). On the other hand, glioma patients who expressed lower levels of Sema3g showed shortened survival (Karayan-Tapon et al., 2008). We observed a parallel pattern in our analysis where *sema3gb* was upregulated at 3 dpl and 14 dpl while being downregulated in GBM. Interestingly, a large number of semaphorin genes were exclusively downregulated at 1 dpl and were not altered at later stages. Several semaphorins including Sema3a, Sema3f, Sema3g and Sema6a have been reported to exert tumor growth-inhibiting activities while several others such as Sema4d and Sema6d have been associated with tumor-promoting functions in various cancer types (Law and Lee, 2012; Angelucci et al., 2019). Thus, detailed functional analyses for individual semaphorins are essential to compare their roles in brain regeneration and brain cancer.

## Conclusion and Future Directions

In conclusion, our comparative analyses of the transcriptomes of the regenerating zebrafish brain at three different regenerative stages with those of two different brain cancers reveal the common and distinctive mechanisms that operate during regeneration and cancer of the brain. Characterization of cellular signals that ensure timely cessation of proliferation, a key step of regeneration, at the correct and controlled termination of regeneration might indeed be exceptionally helpful to identify candidate signals that can stop abnormal proliferative responses to chronic injury or inflammation, stop tumor growth and, perhaps, even direct tumor cells to a regeneration-like route. At this point, the zebrafish represents an excellent model with its organs that show high homology to those of mammals, regenerate and can be induced to develop cancer. Future studies that compare regeneration and cancer using their zebrafish models will not only contribute to our understanding of differential mechanisms of both phenomena but also open new avenues in development of novel anti-cancer therapies. Moreover, an elegant work has presented a comprehensive approach for the DNA methylation-based classification of central nervous system tumors (Capper et al., 2018). Thus, we believe that identification of the genome-wide DNA methylation profiles of the regenerating zebrafish brain and comparison of these cohorts to the human brain tumor classifiers will reinforce our understanding of regulation of brain regeneration mechanisms.

## Data Availability

All datasets have been deposited in ArrayExpress under the link: https://www.ebi.ac.uk/arrayexpress/experiments/E-MTAB-11163/ with the accession number “E-MTAB-11163”.

## References

[B1] Agnihotri S. Burrell K. E. Wolf A. Jalali S. Hawkins C. Rutka J. T. (2013). Glioblastoma, a Brief Review of History, Molecular Genetics, Animal Models and Novel Therapeutic Strategies. Arch. Immunol. Ther. Exp. 61 (1), 25–41. 10.1007/s00005-012-0203-0 23224339

[B2] Anders S. Pyl P. T. Huber W. (2015). HTSeq--a Python Framework to Work with High-Throughput Sequencing Data. Bioinformatics 31 (2), 166–169. 10.1093/bioinformatics/btu638 25260700 PMC4287950

[B3] Andrae J. Gallini R. Betsholtz C. (2008). Role of Platelet-Derived Growth Factors in Physiology and Medicine. Genes Dev. 22 (10), 1276–1312. 10.1101/gad.1653708 18483217 PMC2732412

[B4] Andrews S. (2010). A Quality Control Tool for High Throughput Sequence Data.

[B5] Angelucci C. Lama G. Sica G. (2019). Multifaceted Functional Role of Semaphorins in Glioblastoma. Ijms 20 (9), 2144. 10.3390/ijms20092144 PMC6539029 31052281

[B6] Bao S. Wu Q. McLendon R. E. Hao Y. Shi Q. Hjelmeland A. B. (2006). Glioma Stem Cells Promote Radioresistance by Preferential Activation of the DNA Damage Response. Nature 444 (7120), 756–760. 10.1038/nature05236 17051156

[B7] Baumgart E. V. Barbosa J. S. Bally-Cuif L. Götz M. Ninkovic J. (2012). Stab Wound Injury of the Zebrafish Telencephalon: a Model for Comparative Analysis of Reactive Gliosis. Glia 60 (3), 343–357. 10.1002/glia.22269 22105794

[B8] Beachy P. A. Karhadkar S. S. Berman D. M. (2004). Tissue Repair and Stem Cell Renewal in Carcinogenesis. Nature 432 (7015), 324–331. 10.1038/nature03100 15549094

[B9] Beane W. S. Morokuma J. Lemire J. M. Levin M. (2013). Bioelectric Signaling Regulates Head and Organ Size during Planarian Regeneration. Development (Cambridge, England) 140 (2), 313–322. 10.1242/dev.086900 PMC3597208 23250205

[B10] Benitez J. A. Ma J. D’Antonio M. Boyer A. Camargo M. F. Zanca C. (2017). PTEN Regulates Glioblastoma Oncogenesis through Chromatin-Associated Complexes of DAXX and Histone H3.3. Nat. Commun. 8 (1), 15223. 10.1038/ncomms15223 28497778 PMC5437297

[B11] Boffetta P. Boccia S. Vecchia C. L. (2014). “Distribution, Causes and Prevention of Individual Neoplasms,” in A Quick Guide to Cancer Epidemiology. SpringerBriefs in Cancer Research (Cham: Springer). 10.1007/978-3-319-05068-3_4

[B12] Bondy M. L. Scheurer M. E. Malmer B. Barnholtz-Sloan J. S. Davis F. G. Il'yasova D. (2008). Brain Tumor Epidemiology: Consensus from the Brain Tumor Epidemiology Consortium. Cancer 113 (7 Suppl. l), 1953–1968. 10.1002/cncr.23741 18798534 PMC2861559

[B13] Bonfanti L. Peretto P. (2011). Adult Neurogenesis in Mammals - a Theme with many Variations. Eur. J. Neurosci. 34 (6), 930–950. 10.1111/j.1460-9568.2011.07832.x 21929626

[B14] Brown D. L. Kao W. W.-Y. Greenhalgh D. G. (1997). Apoptosis Down-Regulates Inflammation under the Advancing Epithelial Wound Edge: Delayed Patterns in Diabetes and Improvement with Topical Growth Factors. Surgery 121 (4), 372–380. 10.1016/s0039-6060(97)90306-8 9122866

[B15] Bussmann J. Raz E. (2015). Chemokine‐guided Cell Migration and Motility in Zebrafish Development. EMBO J. 34 (10), 1309–1318. 10.15252/embj.201490105 25762592 PMC4491993

[B16] Capper D. Jones D. T. W. Sill M. Hovestadt V. Schrimpf D. Sturm D. (2018). DNA Methylation-Based Classification of central Nervous System Tumours. Nature 555 (7697), 469–474. 10.1038/nature26000 29539639 PMC6093218

[B17] Carbon S. Ireland A. Mungall C. J. Shu S. Marshall B. Lewis S. (2009). AmiGO: Online Access to Ontology and Annotation Data. Bioinformatics 25 (2), 288–289. 10.1093/bioinformatics/btn615 19033274 PMC2639003

[B18] Carulli D. de Winter F. Verhaagen J. (2021). Semaphorins in Adult Nervous System Plasticity and Disease. Front. Synaptic Neurosci. 13 (20). 10.3389/fnsyn.2021.672891 PMC8148045 34045951

[B19] Chen J. Li Y. Yu T.-S. McKay R. M. Burns D. K. Kernie S. G. (2012). A Restricted Cell Population Propagates Glioblastoma Growth after Chemotherapy. Nature 488 (7412), 522–526. 10.1038/nature11287 22854781 PMC3427400

[B20] Chera S. Ghila L. Dobretz K. Wenger Y. Bauer C. Buzgariu W. (2009). Apoptotic Cells Provide an Unexpected Source of Wnt3 Signaling to Drive hydra Head Regeneration. Developmental Cell 17 (2), 279–289. 10.1016/j.devcel.2009.07.014 19686688

[B21] Coussens L. M. Werb Z. (2002). Inflammation and Cancer. Nature 420 (6917), 860–867. 10.1038/nature01322 12490959 PMC2803035

[B22] Das K. K. Kumar R. (2017). “Pediatric Glioblastoma,” in Glioblastoma. Editor De Vleeschouwer S. (Brisbane, AU: Codon Publications). 29251872

[B23] Demirci Y. Cucun G. Poyraz Y. K. Mohammed S. Heger G. Papatheodorou I. (2020). Comparative Transcriptome Analysis of the Regenerating Zebrafish Telencephalon Unravels a Resource with Key Pathways during Two Early Stages and Activation of Wnt/β-Catenin Signaling at the Early Wound Healing Stage. Front. Cell Dev. Biol. 8, 584604. 10.3389/fcell.2020.584604 33163496 PMC7581945

[B24] Diotel N. Lübke L. Strähle U. Rastegar S. (2020). Common and Distinct Features of Adult Neurogenesis and Regeneration in the Telencephalon of Zebrafish and Mammals. Front. Neurosci. 14 (957). 10.3389/fnins.2020.568930 PMC7538694 33071740

[B25] Diwanji N. Bergmann A. (2018). An Unexpected Friend − ROS in Apoptosis-Induced Compensatory Proliferation: Implications for Regeneration and Cancer. Semin. Cell Developmental Biol. 80, 74–82. 10.1016/j.semcdb.2017.07.004 PMC5756134 28688927

[B26] Duhen T. Ni C. Campbell D. (2014). Identification of a Specific Gene Signature in Human Th1/17 Cells (BA13P.126). J. Immunol. 192 (1 Suppl. ment), 177112.

[B27] Elsaeidi F. Bemben M. A. Zhao X.-F. Goldman D. (2014). Jak/Stat Signaling Stimulates Zebrafish Optic Nerve Regeneration and Overcomes the Inhibitory Actions of Socs3 and Sfpq. J. Neurosci. 34 (7), 2632–2644. 10.1523/JNEUROSCI.3898-13.2014 24523552 PMC3921430

[B28] Fernandes C. Costa A. Osorio L. Lago R. C. Linhares P. Carvalho B. (2017). “Current Standards of Care in Glioblastoma Therapy,” in Glioblastoma. Editor De Vleeschouwer S. (Brisbane, AU: Codon Publications). 29251860

[B29] Flier J. S. Underhill L. H. Dvorak H. F. (1986). Tumors: Wounds that Do Not Heal. N. Engl. J. Med. 315 (26), 1650–1659. 10.1056/NEJM198612253152606 3537791

[B30] Fresegna D. Bullitta S. Musella A. Rizzo F. R. De Vito F. Guadalupi L. (2020). Re-Examining the Role of TNF in MS Pathogenesis and Therapy. Cells 9 (10), 2290. 10.3390/cells9102290 PMC7602209 33066433

[B31] Fuller-Carter P. I. Carter K. W. Anderson D. Harvey A. R. Giles K. M. Rodger J. (2015). Integrated Analyses of Zebrafish miRNA and mRNA Expression Profiles Identify miR-29b and miR-223 as Potential Regulators of Optic Nerve Regeneration. BMC Genomics 16, 591. 10.1186/s12864-015-1772-1 26265132 PMC4534052

[B32] Gargioli C. Slack J. M. W. (2004). Cell Lineage Tracing duringXenopustail Regeneration. Development 131 (11), 2669–2679. 10.1242/dev.01155 15148301

[B33] Gauron C. Rampon C. Bouzaffour M. Ipendey E. Teillon J. Volovitch M. (2013). Sustained Production of ROS Triggers Compensatory Proliferation and Is Required for Regeneration to Proceed. Sci. Rep. 3, 2084. 10.1038/srep02084 23803955 PMC3694286

[B34] Ghosh S. Hui S. P. (2016). Regeneration of Zebrafish CNS: Adult Neurogenesis. Neural Plasticity 2016, 1–21. 10.1155/2016/5815439 PMC4921647 27382491

[B35] Gilbert C. A. Ross A. H. (2009). Cancer Stem Cells: Cell Culture, Markers, and Targets for New Therapies. J. Cell. Biochem. 108 (5), 1031–1038. 10.1002/jcb.22350 19760641 PMC2909872

[B36] Gourain V. Armant O. Lübke L. Diotel N. Rastegar S. Strähle U. (2021). Multi-Dimensional Transcriptome Analysis Reveals Modulation of Cholesterol Metabolism as Highly Integrated Response to Brain Injury. Front. Neurosci. 15 (543). 10.3389/fnins.2021.671249 PMC8162057 34054419

[B37] Grandel H. Kaslin J. Ganz J. Wenzel I. Brand M. (2006). Neural Stem Cells and Neurogenesis in the Adult Zebrafish Brain: Origin, Proliferation Dynamics, Migration and Cell Fate. Developmental Biol. 295 (1), 263–277. 10.1016/j.ydbio.2006.03.040 16682018

[B38] Guerin D. J. Kha C. X. Tseng K. A.-S. (2021). From Cell Death to Regeneration: Rebuilding after Injury. Front. Cell Dev. Biol. 9 (547). 10.3389/fcell.2021.655048 PMC8012889 33816506

[B39] Guo S.-K. Shen M.-F. Yao H.-W. Liu Y.-S. (2018). Enhanced Expression of TGFBI Promotes the Proliferation and Migration of Glioma Cells. Cell Physiol Biochem 49 (3), 1138–1150. 10.1159/000493293 30196284

[B40] Gurtner G. C. Werner S. Barrandon Y. Longaker M. T. (2008). Wound Repair and Regeneration. Nature 453 (7193), 314–321. 10.1038/nature07039 18480812

[B41] Haddow A. (1972). Molecular Repair, Wound Healing, and Carcinogenesis: Tumor Production a Possible Overhealing? Adv. Cancer Res. 16, 181–234. 10.1016/s0065-230x(08)60341-3 4563044

[B42] Hagedorn M. Siegfried G. Hooks K. B. Khatib A.-M. (2016). Integration of Zebrafish Fin Regeneration Genes with Expression Data of Human Tumors In Silico Uncovers Potential Novel Melanoma Markers. Oncotarget 7 (44), 71567–71579. 10.18632/oncotarget.12257 27689402 PMC5342102

[B43] Hamalainen H. Zhou H. Chou W. Hashizume H. Heller R. Lahesmaa R. (2001). Distinct Gene Expression Profiles of Human Type 1 and Type 2 T Helper Cells. Genome Biol. 2 (7), research00221. 10.1186/gb-2001-2-7-research0022 PMC55319 11516335

[B44] Hanif F. Muzaffar K. Perveen K. Malhi S. M. Simjee Sh. U. (2017). Glioblastoma Multiforme: A Review of its Epidemiology and Pathogenesis through Clinical Presentation and Treatment. Asian Pac. J. Cancer Prev. 18 (1), 3–9. 10.22034/APJCP.2017.18.1.3 28239999 PMC5563115

[B45] He Q. Li Z. (2021). The Dysregulated Expression and Functional Effect of CaMK2 in Cancer. Cancer Cell Int 21 (1), 326. 10.1186/s12935-021-02030-7 34193145 PMC8243487

[B46] Hemmati H. D. Nakano I. Lazareff J. A. Masterman-Smith M. Geschwind D. H. Bronner-Fraser M. (2003). Cancerous Stem Cells Can Arise from Pediatric Brain Tumors. Proc. Natl. Acad. Sci. 100 (25), 15178–15183. 10.1073/pnas.2036535100 14645703 PMC299944

[B47] Hirakata C. Lima K. De Almeida B. De Miranda L. Florêncio K. Furtado L. (2021). Targeting Glioma Cells by Antineoplastic Activity of Reversine. Oncol. Lett. 22 (2), 610. 10.3892/ol.2021.12871 34188712 PMC8227489

[B48] Huang D. W. Sherman B. T. Lempicki R. A. (2009). Systematic and Integrative Analysis of Large Gene Lists Using DAVID Bioinformatics Resources. Nat. Protoc. 4 (1), 44–57. 10.1038/nprot.2008.211 19131956

[B49] Idilli A. Precazzini F. Mione M. Anelli V. (2017). Zebrafish in Translational Cancer Research: Insight into Leukemia, Melanoma, Glioma and Endocrine Tumor Biology. Genes 8 (9), 236. 10.3390/genes8090236 PMC5615369 28930163

[B50] Imayoshi I. Kageyama R. (2011). The Role of Notch Signaling in Adult Neurogenesis. Mol. Neurobiol. 44 (1), 7–12. 10.1007/s12035-011-8186-0 21541768

[B51] Iribarne M. (2021). Inflammation Induces Zebrafish Regeneration. Neural Regen. Res. 16 (9), 1693–1701. 10.4103/1673-5374.306059 33510057 PMC8328752

[B52] Jaerve A. Müller H. W. (2012). Chemokines in CNS Injury and Repair. Cell Tissue Res 349 (1), 229–248. 10.1007/s00441-012-1427-3 22700007

[B53] Jeansson M. Gawlik A. Anderson G. Li C. Kerjaschki D. Henkelman M. (2011). Angiopoietin-1 Is Essential in Mouse Vasculature during Development and in Response to Injury. J. Clin. Invest. 121 (6), 2278–2289. 10.1172/JCI46322 21606590 PMC3104773

[B54] Johansson F. K. Göransson H. Westermark B. (2005). Expression Analysis of Genes Involved in Brain Tumor Progression Driven by Retroviral Insertional Mutagenesis in Mice. Oncogene 24 (24), 3896–3905. 10.1038/sj.onc.1208553 15750623

[B55] Jones C. Karajannis M. A. Jones D. T. W. Kieran M. W. Monje M. Baker S. J. (2017). Pediatric High-Grade Glioma: Biologically and Clinically in Need of New Thinking. Neuro Oncol. 19 (2), now101–161. 10.1093/neuonc/now101 PMC5464243 27282398

[B56] Jopling C. Sleep E. Raya M. Martí M. Raya A. Belmonte J. C. I. (2010). Zebrafish Heart Regeneration Occurs by Cardiomyocyte Dedifferentiation and Proliferation. Nature 464 (7288), 606–609. 10.1038/nature08899 20336145 PMC2846535

[B57] Jurisch‐Yaksi N. Yaksi E. Kizil C. (2020). Radial Glia in the Zebrafish Brain: Functional, Structural, and Physiological Comparison with the Mammalian Glia. Glia 68 (12), 2451–2470. 10.1002/glia.23849 32476207

[B58] Kanazawa M. Hatakeyama M. Ninomiya I. (2020). Angiogenesis and Neuronal Remodeling after Ischemic Stroke. Neural Regen. Res. 15 (1), 16–19. 10.4103/1673-5374.264442 31535636 PMC6862417

[B59] Kanazawa M. Miura M. Toriyabe M. Koyama M. Hatakeyama M. Ishikawa M. (2017). Microglia Preconditioned by Oxygen-Glucose Deprivation Promote Functional Recovery in Ischemic Rats. Sci. Rep. 7 (1), 42582. 10.1038/srep42582 28195185 PMC5307390

[B60] Karayan-Tapon L. Wager M. Guilhot J. Levillain P. Marquant C. Clarhaut J. (2008). Semaphorin, Neuropilin and VEGF Expression in Glial Tumours: SEMA3G, a Prognostic Marker? Br. J. Cancer 99 (7), 1153–1160. 10.1038/sj.bjc.6604641 18781179 PMC2567090

[B61] Kaslin J. Kroehne V. Ganz J. Hans S. Brand M. (2017). Distinct Roles of Neuroepithelial-like and Radial Glia-like Progenitor Cells in Cerebellar Regeneration. Development 144 (8), 1462–1471. 10.1242/dev.144907 28289134

[B62] Katoh M. (2002). Expression of Human SOX7 in normal Tissues and Tumors. Int. J. Mol. Med. 9 (4), 363–368. 10.3892/ijmm.9.4.363 11891528

[B63] Kha C. X. Son P. H. Lauper J. Tseng K. A.-S. (2018). A Model for Investigating Developmental Eye Repair in *Xenopus laevis* . Exp. Eye Res. 169, 38–47. 10.1016/j.exer.2018.01.007 29357285

[B64] Kim D. Langmead B. Salzberg S. L. (2015). HISAT: a Fast Spliced Aligner with Low Memory Requirements. Nat. Methods 12 (4), 357–360. 10.1038/nmeth.3317 25751142 PMC4655817

[B65] Kim E. Kim M. Woo D.-H. Shin Y. Shin J. Chang N. (2013). Phosphorylation of EZH2 Activates STAT3 Signaling via STAT3 Methylation and Promotes Tumorigenicity of Glioblastoma Stem-like Cells. Cancer Cell 23 (6), 839–852. 10.1016/j.ccr.2013.04.008 23684459 PMC4109796

[B66] Kim I.-K. Kim K. Lee E. Oh D. S. Park C. S. Park S. (2018). Sox7 Promotes High-Grade Glioma by Increasing VEGFR2-Mediated Vascular Abnormality. J. Exp. Med. 215 (3), 963–983. 10.1084/jem.20170123 29444818 PMC5839752

[B67] Kizil C. Dudczig S. Kyritsis N. Machate A. Blaesche J. Kroehne V. (2012a). The Chemokine Receptor Cxcr5 Regulates the Regenerative Neurogenesis Response in the Adult Zebrafish Brain. Neural Dev. 7, 27. 10.1186/1749-8104-7-27 22824261 PMC3441421

[B68] Kizil C. Kaslin J. Kroehne V. Brand M. (2012b). Adult Neurogenesis and Brain Regeneration in Zebrafish. Devel Neurobio 72 (3), 429–461. 10.1002/dneu.20918 21595047

[B69] Kolde R. (2012). Pheatmap: Pretty Heatmaps. R. Package Version 1 (2), 726.

[B70] Krafts K. P. (2010). Tissue Repair. Organogenesis 6 (4), 225–233. 10.4161/org.6.4.12555 21220961 PMC3055648

[B71] Kriegstein A. Alvarez-Buylla A. (2009). The Glial Nature of Embryonic and Adult Neural Stem Cells. Annu. Rev. Neurosci. 32, 149–184. 10.1146/annurev.neuro.051508.135600 19555289 PMC3086722

[B72] Kroehne V. Freudenreich D. Hans S. Kaslin J. Brand M. (2011). Regeneration of the Adult Zebrafish Brain from Neurogenic Radial Glia-type Progenitors. Development 138 (22), 4831–4841. 10.1242/dev.072587 22007133

[B73] Kyritsis N. Kizil C. Zocher S. Kroehne V. Kaslin J. Freudenreich D. (2012). Acute Inflammation Initiates the Regenerative Response in the Adult Zebrafish Brain. Science 338 (6112), 1353–1356. 10.1126/science.1228773 23138980

[B74] Ladomersky E. Scholtens D. M. Kocherginsky M. Hibler E. A. Bartom E. T. Otto-Meyer S. (2019). The Coincidence between Increasing Age, Immunosuppression, and the Incidence of Patients with Glioblastoma. Front. Pharmacol. 10, 200. 10.3389/fphar.2019.00200 30971917 PMC6446059

[B75] Lange C. Rost F. Machate A. Reinhardt S. Lesche M. Weber A. (2020). Single Cell Sequencing of Radial Glia Progeny Reveals Diversity of Newborn Neurons in the Adult Zebrafish Brain. Development 147 (1), 1855951. 10.1242/dev.185595 PMC6983714 31908317

[B76] Langfelder P. Horvath S. (2008). WGCNA: an R Package for Weighted Correlation Network Analysis. BMC Bioinformatics 9, 559. 10.1186/1471-2105-9-559 19114008 PMC2631488

[B77] Law J. W. S. Lee A. Y. W. (2012). The Role of Semaphorins and Their Receptors in Gliomas. J. Signal Transduction 2012, 1–14. 10.1155/2012/902854 PMC3461631 23050142

[B78] Li X. Nie S. Lv Z. Ma L. Song Y. Hu Z. (2021). Overexpression of Annexin A2 Promotes Proliferation by Forming a Glypican 1/c-Myc Positive Feedback Loop: Prognostic Significance in Human Glioma. Cell Death Dis 12 (3), 261. 10.1038/s41419-021-03547-5 33712571 PMC7954792

[B79] Li X. Wu C. Chen N. Gu H. Yen A. Cao L. (2016). PI3K/Akt/mTOR Signaling Pathway and Targeted Therapy for Glioblastoma. Oncotarget 7 (22), 33440–33450. 10.18632/oncotarget.7961 26967052 PMC5078108

[B80] Lindsey B. W. Douek A. M. Loosli F. Kaslin J. (2017). A Whole Brain Staining, Embedding, and Clearing Pipeline for Adult Zebrafish to Visualize Cell Proliferation and Morphology in 3-Dimensions. Front. Neurosci. 11, 750. 10.3389/fnins.2017.00750 29386991 PMC5776138

[B81] Liu H. Yan Z.-Q. Li B. Yin S.-Y. Sun Q. Kou J.-J. (2014). Reduced Expression of SOX7 in Ovarian Cancer: a Novel Tumor Suppressor through the Wnt/β-Catenin Signaling Pathway. J. Ovarian Res. 7, 87. 10.1186/s13048-014-0087-1 25297608 PMC4172779

[B82] Louis D. N. Perry A. Reifenberger G. von Deimling A. Figarella-Branger D. Cavenee W. K. (2016). The 2016 World Health Organization Classification of Tumors of the Central Nervous System: a Summary. Acta Neuropathol. 131 (6), 803–820. 10.1007/s00401-016-1545-1 27157931

[B83] Louis D. N. Perry A. Wesseling P. Brat D. J. Cree I. A. Figarella-Branger D. (2021). The 2021 WHO Classification of Tumors of the Central Nervous System: a Summary. Neuro Oncol. 23 (8), 1231–1251. 10.1093/neuonc/noab106 34185076 PMC8328013

[B84] Love M. I. Huber W. Anders S. (2014). Moderated Estimation of Fold Change and Dispersion for RNA-Seq Data with DESeq2. Genome Biol. 15 (12), 550. 10.1186/s13059-014-0550-8 25516281 PMC4302049

[B85] Lu H.-J. Yan J. Jin P.-Y. Zheng G.-H. Zhang H.-L. Bai M. (2018). Mechanism of MicroRNA-708 Targeting BAMBI in Cell Proliferation, Migration, and Apoptosis in Mice with Melanoma via the Wnt and TGF-β Signaling Pathways. Technol. Cancer Res. Treat. 17, 153303461875678. 10.1177/1533034618756784 PMC5826012 29466930

[B86] Lymbouridou R. Soufla G. Chatzinikola A. M. Vakis A. Spandidos D. A. (2009). Down-regulation of K-Ras and H-Ras in Human Brain Gliomas. Eur. J. Cancer 45 (7), 1294–1303. 10.1016/j.ejca.2008.12.028 19179066

[B87] Magrassi L. Conti L. Lanterna A. Zuccato C. Marchionni M. Cassini P. (2005). Shc3 Affects Human High-Grade Astrocytomas Survival. Oncogene 24 (33), 5198–5206. 10.1038/sj.onc.1208708 15870690

[B88] Mao H. Lebrun D. G. Yang J. Zhu V. F. Li M. (2012). Deregulated Signaling Pathways in Glioblastoma Multiforme: Molecular Mechanisms and Therapeutic Targets. Cancer Invest. 30 (1), 48–56. 10.3109/07357907.2011.630050 22236189 PMC3799884

[B89] Marín-Juez R. Marass M. Gauvrit S. Rossi A. Lai S.-L. Materna S. C. (2016). Fast Revascularization of the Injured Area Is Essential to Support Zebrafish Heart Regeneration. Proc. Natl. Acad. Sci. USA 113 (40), 11237–11242. 10.1073/pnas.1605431113 27647901 PMC5056108

[B90] Marques I. J. Lupi E. Mercader N. (2019). Model Systems for Regeneration: Zebrafish. Development 146 (18). 10.1242/dev.167692 31540899

[B91] Méndez O. Zavadil J. Esencay M. Lukyanov Y. Santovasi D. Wang S.-C. (2010). Knock Down of HIF-1α in Glioma Cells Reduces Migration *In Vitro* and Invasion *In Vivo* and Impairs Their Ability to Form Tumor Spheres. Mol. Cancer 9, 133. 10.1186/1476-4598-9-133 20515450 PMC2896954

[B92] Merchant T. E. Pollack I. F. Loeffler J. S. (2010). Brain Tumors across the Age Spectrum: Biology, Therapy, and Late Effects. Semin. Radiat. Oncol. 20 (1), 58–66. 10.1016/j.semradonc.2009.09.005 19959032 PMC3529408

[B93] Messali A. Villacorta R. Hay J. W. (2014). A Review of the Economic burden of Glioblastoma and the Cost Effectiveness of Pharmacologic Treatments. Pharmacoeconomics 32 (12), 1201–1212. 10.1007/s40273-014-0198-y 25085219

[B94] Morata G. Shlevkov E. Pérez-Garijo A. (2011). Mitogenic Signaling from Apoptotic Cells in Drosophila. Dev. Growth Differ. 53 (2), 168–176. 10.1111/j.1440-169X.2010.01225.x 21338343

[B95] Nasser M. M. Mehdipour P. (2018). Exploration of Involved Key Genes and Signaling Diversity in Brain Tumors. Cell Mol Neurobiol 38 (2), 393–419. 10.1007/s10571-017-0498-9 28493234 PMC11481865

[B96] National Cancer Institute (2020). Genomic Data Commons Data Portal. Bethesda, Maryland: National Cancer Institute.

[B97] Oh K.-Y. Hong K.-O. Huh Y.-S. Lee J.-I. Hong S.-D. (2017). Decreased Expression of SOX7 Induces Cell Proliferation and Invasion and Correlates with Poor Prognosis in Oral Squamous Cell Carcinoma. J. Oral Pathol. Med. 46 (9), 752–758. 10.1111/jop.12566 28266739

[B98] Ohgaki H. Kleihues P. (2007). Genetic Pathways to Primary and Secondary Glioblastoma. Am. J. Pathol. 170 (5), 1445–1453. 10.2353/ajpath.2007.070011 17456751 PMC1854940

[B99] Ohka F. Natsume A. Wakabayashi T. (2012). Current Trends in Targeted Therapies for Glioblastoma Multiforme. Neurol. Res. Int. 2012, 1–13. 10.1155/2012/878425 PMC3317017 22530127

[B100] Ostrom Q. T. Gittleman H. Farah P. Ondracek A. Chen Y. Wolinsky Y. (2013). CBTRUS Statistical Report: Primary Brain and central Nervous System Tumors Diagnosed in the United States in 2006-2010. Neuro-Oncology 15 (Suppl. 2), ii1–ii56. 10.1093/neuonc/not151 24137015 PMC3798196

[B101] Oviedo N. J. Beane W. S. (2009). Regeneration: The Origin of Cancer or a Possible Cure? Semin. Cell Developmental Biol. 20 (5), 557–564. 10.1016/j.semcdb.2009.04.005 PMC2706275 19427247

[B102] Pellettieri J. Fitzgerald P. Watanabe S. Mancuso J. Green D. R. Sánchez Alvarado A. (2010). Cell Death and Tissue Remodeling in Planarian Regeneration. Developmental Biol. 338 (1), 76–85. 10.1016/j.ydbio.2009.09.015 PMC2835816 19766622

[B103] Peng G. Wang Y. Ge P. Bailey C. Zhang P. Zhang D. (2021). The HIF1α-PDGFD-Pdgfrα axis Controls Glioblastoma Growth at Normoxia/mild-Hypoxia and Confers Sensitivity to Targeted Therapy by Echinomycin. J. Exp. Clin. Cancer Res. 40 (1), 278. 10.1186/s13046-021-02082-7 34470658 PMC8411541

[B104] Pereira J. D. Sansom S. N. Smith J. Dobenecker M.-W. Tarakhovsky A. Livesey F. J. (2010). Ezh2, the Histone Methyltransferase of PRC2, Regulates the Balance between Self-Renewal and Differentiation in the Cerebral Cortex. Proc. Natl. Acad. Sci. 107 (36), 15957–15962. 10.1073/pnas.1002530107 20798045 PMC2936600

[B105] Pierce G. F. Mustoe T. A. Altrock B. W. Deuel T. F. Thomason A. (1991). Role of Platelet-Derived Growth Factor in Wound Healing. J. Cell. Biochem. 45 (4), 319–326. 10.1002/jcb.240450403 2045423

[B106] Prasad G. Haas-Kogan D. A. (2009). Radiation-induced Gliomas. Expert Rev. Neurotherapeutics 9 (10), 1511–1517. 10.1002/pmic.20080080210.1586/ern.09.98 PMC3830919 19831840

[B107] Rahaman S. O. Harbor P. C. Chernova O. Barnett G. H. Vogelbaum M. A. Haque S. J. (2002). Inhibition of Constitutively Active Stat3 Suppresses Proliferation and Induces Apoptosis in Glioblastoma Multiforme Cells. Oncogene 21 (55), 8404–8413. 10.1038/sj.onc.1206047 12466961

[B108] Raphael I. Nalawade S. Eagar T. N. Forsthuber T. G. (2015). T Cell Subsets and Their Signature Cytokines in Autoimmune and Inflammatory Diseases. Cytokine 74 (1), 5–17. 10.1016/j.cyto.2014.09.011 25458968 PMC4416069

[B109] Ruan L. Wang B. ZhuGe Q. Jin K. (2015). Coupling of Neurogenesis and Angiogenesis after Ischemic Stroke. Brain Res. 1623, 166–173. 10.1016/j.brainres.2015.02.042 25736182 PMC4552615

[B110] Schäfer M. Werner S. (2008). Cancer as an Overhealing Wound: an Old Hypothesis Revisited. Nat. Rev. Mol. Cell Biol 9 (8), 628–638. 10.1038/nrm2455 18628784

[B111] Schmidt R. Beil T. Strähle U. Rastegar S. (2014). Stab Wound Injury of the Zebrafish Adult Telencephalon: a Method to Investigate Vertebrate Brain Neurogenesis and Regeneration. JoVE 90, e51753. 10.3791/51753 PMC4692347 25146302

[B112] Schwartzbaum J. A. Fisher J. L. Aldape K. D. Wrensch M. (2006). Epidemiology and Molecular Pathology of Glioma. Nat. Rev. Neurol. 2 (9), 494–503. quiz 491 p following 516. 10.1038/ncpneuro0289 16932614

[B113] Sherry M. M. Reeves A. Wu J. K. Cochran B. H. (2009). STAT3 Is Required for Proliferation and Maintenance of Multipotency in Glioblastoma Stem Cells. Stem Cells 27 (10), 2383–2392. 10.1002/stem.185 19658181 PMC4391626

[B114] Singh S. K. Hawkins C. Clarke I. D. Squire J. A. Bayani J. Hide T. (2004). Identification of Human Brain Tumour Initiating Cells. Nature 432 (7015), 396–401. 10.1038/nature03128 15549107

[B115] Smedley D. Haider S. Ballester B. Holland R. London D. Thorisson G. (2009). BioMart - Biological Queries Made Easy. BMC Genomics 10, 22. 10.1186/1471-2164-10-22 19144180 PMC2649164

[B116] Soengas M. S. Gerald W. L. Cordon-Cardo C. Lazebnik Y. Lowe S. W. (2006). Apaf-1 Expression in Malignant Melanoma. Cell Death Differ 13 (2), 352–353. 10.1038/sj.cdd.4401755 16110320

[B117] Sonar S. Lal G. (2015). Role of Tumor Necrosis Factor Superfamily in Neuroinflammation and Autoimmunity. Front. Immunol. 6, 364. 10.3389/fimmu.2015.00364 26257732 PMC4507150

[B118] Speidel D. (2015). The Role of DNA Damage Responses in P53 Biology. Arch. Toxicol. 89 (4), 501–517. 10.1007/s00204-015-1459-z 25618545

[B119] Stern C. D. (2000). Conrad H. Waddington's Contributions to Avian and Mammalian Development, 1930-1940. Int. J. Dev. Biol. 44 (1), 15–22. 10761841

[B120] Stocum D. L. (2019). Nerves and Proliferation of Progenitor Cells in Limb Regeneration. Develop Neurobiol. 79 (5), 468–478. 10.1002/dneu.22643 30303627

[B121] Stovall D. B. Wan M. Miller L. D. Cao P. Maglic D. Zhang Q. (2013). The Regulation of SOX7 and its Tumor Suppressive Role in Breast Cancer. Am. J. Pathol. 183 (5), 1645–1653. 10.1016/j.ajpath.2013.07.025 24012678 PMC3814686

[B122] Stupp R. Mason W. P. van den Bent M. J. Weller M. Fisher B. Taphoorn M. J. B. (2005). Radiotherapy Plus Concomitant and Adjuvant Temozolomide for Glioblastoma. N. Engl. J. Med. 352 (10), 987–996. 10.1056/NEJMoa043330 15758009

[B123] Suvà M.-L. Riggi N. Janiszewska M. Radovanovic I. Provero P. Stehle J.-C. (2009). EZH2 Is Essential for Glioblastoma Cancer Stem Cell Maintenance. Cancer Res. 69 (24), 9211–9218. 10.1158/0008-5472.can-09-1622 19934320

[B124] Tamimi A. F. Juweid M. (2017). “Epidemiology and Outcome of Glioblastoma,” in Glioblastoma. Editor De Vleeschouwer S. (Brisbane, AU: Codon Publications). 29251870

[B125] Tan C. Lu N.-N. Wang C.-K. Chen D.-Y. Sun N.-H. Lyu H. (2019). Endothelium-Derived Semaphorin 3G Regulates Hippocampal Synaptic Structure and Plasticity via Neuropilin-2/PlexinA4. Neuron 101 (5), 920–937. e913. 10.1016/j.neuron.2018.12.036 30685224

[B126] Tanase C. Albulescu R. Codrici E. Calenic B. Popescu I. D. Mihai S. (2015). Decreased Expression of APAF-1 and Increased Expression of Cathepsin B in Invasive Pituitary Adenoma. Ott 8, 81–90. 10.2147/OTT.S70886 PMC4278787 25565868

[B127] Tsarouchas T. M. Wehner D. Cavone L. Munir T. Keatinge M. Lambertus M. (2018). Dynamic Control of Proinflammatory Cytokines Il-1β and Tnf-α by Macrophages in Zebrafish Spinal Cord Regeneration. Nat. Commun. 9 (1), 4670. 10.1038/s41467-018-07036-w 30405119 PMC6220182

[B128] Tseng A.-S. Adams D. S. Qiu D. Koustubhan P. Levin M. (2007). Apoptosis Is Required during Early Stages of Tail Regeneration in *Xenopus laevis* . Developmental Biol. 301 (1), 62–69. 10.1016/j.ydbio.2006.10.048 PMC3136124 17150209

[B129] Venkatesan S. Lamfers M. L. Dirven C. M. Leenstra S. (2016). Genetic Biomarkers of Drug Response for Small-Molecule Therapeutics Targeting the RTK/Ras/PI3K, P53 or Rb Pathway in Glioblastoma. CNS Oncol. 5 (2), 77–90. 10.2217/cns-2015-0005 26986934 PMC6047437

[B130] Verkhratsky A. Butt A. (2013). “General Pathophysiology of Neuroglia,” in Glial Physiology and Pathophysiology. Editors Verkhratsky A. Butt A. (Hoboken, NJ: Wiley), 431–450.

[B131] Viotti J. Duplan E. Caillava C. Condat J. Goiran T. Giordano C. (2014). Glioma Tumor Grade Correlates with Parkin Depletion in Mutant P53-Linked Tumors and Results from Loss of Function of P53 Transcriptional Activity. Oncogene 33 (14), 1764–1775. 10.1038/onc.2013.124 23644658

[B132] Waddington C. H. (1935). Cancer and the Theory of Organisers. Nature 135, 606–608. 10.1038/135606a0

[B133] Walter W. Sánchez-Cabo F. Ricote M. (2015). GOplot: an R Package for Visually Combining Expression Data with Functional Analysis: Fig. 1. Bioinformatics 31 (17), 2912–2914. 10.1093/bioinformatics/btv300 25964631

[B134] Wang X. Pei Z. Hossain A. Bai Y. Chen G. (2021). Transcription Factor-Based Gene Therapy to Treat Glioblastoma through Direct Neuronal Conversion. Cancer Biol. Med. 18, 860–874. 10.20892/j.issn.2095-3941.2020.0499 PMC8330525 33755378

[B135] Wang Y. Jin K. Mao X. O. Xie L. Banwait S. Marti H. H. (2007). VEGF-overexpressing Transgenic Mice Show Enhanced post-ischemic Neurogenesis and Neuromigration. J. Neurosci. Res. 85 (4), 740–747. 10.1002/jnr.21169 17243175

[B136] Watzlawik J. O. Warrington A. E. Rodriguez M. (2013). PDGF Is Required for Remyelination-Promoting IgM Stimulation of Oligodendrocyte Progenitor Cell Proliferation. PLOS ONE 8 (2), e55149. 10.1371/journal.pone.0055149 23383310 PMC3562326

[B137] Webster H. d. F. (1997). Growth Factors and Myelin Regeneration in Multiple Sclerosis. Mult. Scler. 3 (2), 113–120. 10.1177/135245859700300210 9291164

[B138] White R. M. Zon L. I. (2008). Melanocytes in Development, Regeneration, and Cancer. Cell Stem Cell 3 (3), 242–252. 10.1016/j.stem.2008.08.005 18786412

[B139] Wickham H. (2016). Ggplot2: Elegant Graphics for Data Analysis. New York: Springer-Verlag.

[B140] Wilson S. E. Chaurasia S. S. Medeiros F. W. (2007). Apoptosis in the Initiation, Modulation and Termination of the Corneal Wound Healing Response. Exp. Eye Res. 85 (3), 305–311. 10.1016/j.exer.2007.06.009 17655845 PMC2039895

[B141] Wu Y.-S. Chen S.-N. (2014). Apoptotic Cell: Linkage of Inflammation and Wound Healing. Front. Pharmacol. 5, 1. 10.3389/fphar.2014.00001 24478702 PMC3896898

[B142] Xiong D. D. Xu W. Q. He R. Q. Dang Y. W. Chen G. Luo D. Z. (2019). In�silico Analysis Identified miRNA-based T-herapeutic A-gents against G-lioblastoma M-ultiforme. Oncol. Rep. 41 (4), 2194–2208. 10.3892/or.2019.7022 30816530 PMC6412522

[B143] Xu C. Hasan S. S. Schmidt I. Rocha S. F. Pitulescu M. E. Bussmann J. (2014). Arteries Are Formed by Vein-Derived Endothelial Tip Cells. Nat. Commun. 5, 5758. 10.1038/ncomms6758 25502622 PMC4275597

[B144] Zacchetti A. van Garderen E. Teske E. Nederbragt H. Dierendonck J. H. Rutteman G. R. (2003). Validation of the Use of Proliferation Markers in Canine Neoplastic and Non-neoplastic Tissues: Comparison of KI-67 and Proliferating Cell Nuclear Antigen (PCNA) Expression versus *In Vivo* Bromodeoxyuridine Labelling by Immunohistochemistry. APMIS 111 (3), 430–438. 10.1034/j.1600-0463.2003.t01-1-1110208.x 12752223

[B145] Zambusi A. Ninkovic J. (2020). Regeneration of the central Nervous System-Principles from Brain Regeneration in Adult Zebrafish. Wjsc 12 (1), 8–24. 10.4252/wjsc.v12.i1.8 32110272 PMC7031763

[B146] Zhang J. Ji F. Liu Y. Lei X. Li H. Ji G. (2014). Ezh2 Regulates Adult Hippocampal Neurogenesis and Memory. J. Neurosci. 34 (15), 5184–5199. 10.1523/JNEUROSCI.4129-13.2014 24719098 PMC6609005

[B147] Zhang J. Jiao J. (2015). Molecular Biomarkers for Embryonic and Adult Neural Stem Cell and Neurogenesis. Biomed. Res. Int. 2015, 1–14. 10.1155/2015/727542 PMC4569757 26421301

[B148] Zhang Y. Dube C. Gibert M. Jr. Cruickshanks N. Wang B. Coughlan M. (2018). The P53 Pathway in Glioblastoma. Cancers 10 (9), 297. 10.3390/cancers10090297 PMC6162501 30200436

[B149] Zhao T. Yang H. Tian Y. Xie Q. Lu Y. Wang Y. (2016). SOX7 Is Associated with the Suppression of Human Glioma by HMG-Box Dependent Regulation of Wnt/β-Catenin Signaling. Cancer Lett. 375 (1), 100–107. 10.1016/j.canlet.2016.02.044 26944317

[B150] Zhou B.-B. S. Zhang H. Damelin M. Geles K. G. Grindley J. C. Dirks P. B. (2009). Tumour-initiating Cells: Challenges and Opportunities for Anticancer Drug Discovery. Nat. Rev. Drug Discov. 8 (10), 806–823. 10.1038/nrd2137 19794444

[B151] Zoncu R. Efeyan A. Sabatini D. M. (2011). mTOR: from Growth Signal Integration to Cancer, Diabetes and Ageing. Nat. Rev. Mol. Cell Biol 12 (1), 21–35. 10.1038/nrm3025 21157483 PMC3390257

