# Supplementary material for: Brain Regeneration Resembles Brain Cancer at Its Early Wound Healing Stage and Diverges From Cancer Later at Its Proliferation and Differentiation Stages
Source: Front Cell Dev Biol. 2022 Feb 10;10:813314. doi: 10.3389/fcell.2022.813314 (PMC8868567; doi:10.3389/fcell.2022.813314)
Supplement: Supplementary file 11 [file DataSheet1.docx]

**Supplementary Figures for Demirci et al.**

**
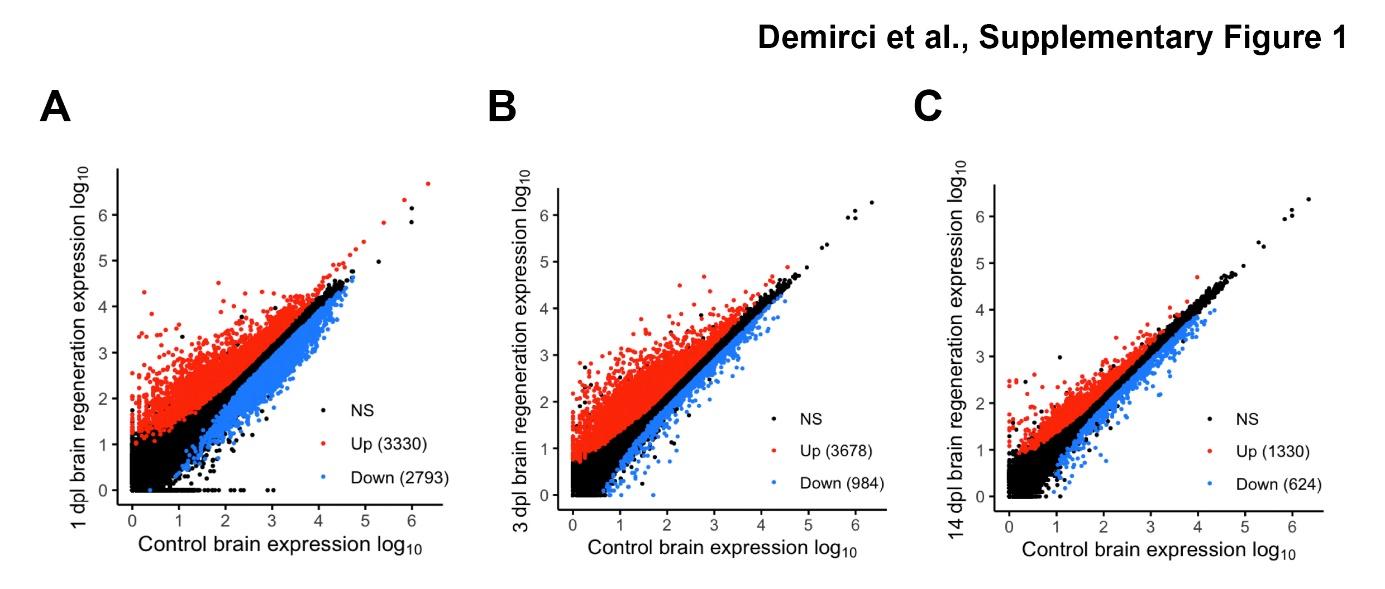
**

**Supplementary Figure 1.** **Transcriptomic profiling of zebrafish brain regeneration.** Scatterplots showing the numbers of upregulated (Up, red) and downregulated (Down, blue) genes at (A) 1 dpl (n=5), (B) 3 dpl (n=4) and (C) 14 dpl (n=4) of brain regeneration compared to unlesioned brain (n=5). dpl: days post-lesion, NS: non-significant.


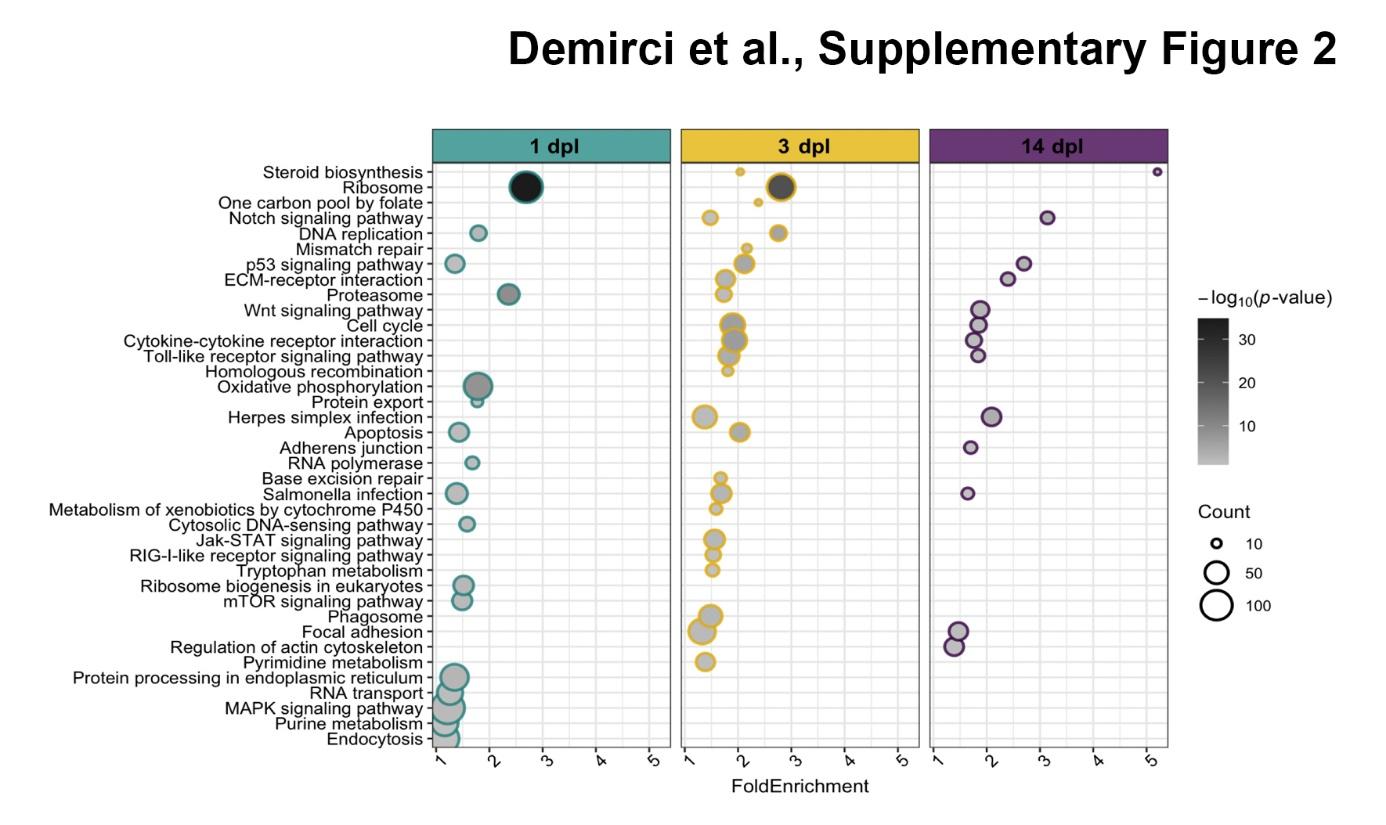
**Supplementary Figure 2. KEGG pathways enriched at three different stages of zebrafish brain regeneration determined by using all DEGs.** DAVID was used to show the most significantly enriched KEGG pathways based on transcriptional changes at 1 dpl, 3 dpl and 14 dpl as compared to the unlesioned brain. All DEGs (1 dpl: 6123, 3 dpl: 4662, 14 dpl: 1954) were used for the analysis. The heatmap’s scale shows negative log_10_ of EASE p-values for all significantly enriched GO terms. dpl: days post-lesion, DAVID: Database for Annotation, Visualization and Integrated Discovery, KEGG: Kyoto Encyclopedia of Genes and Genomes.

**
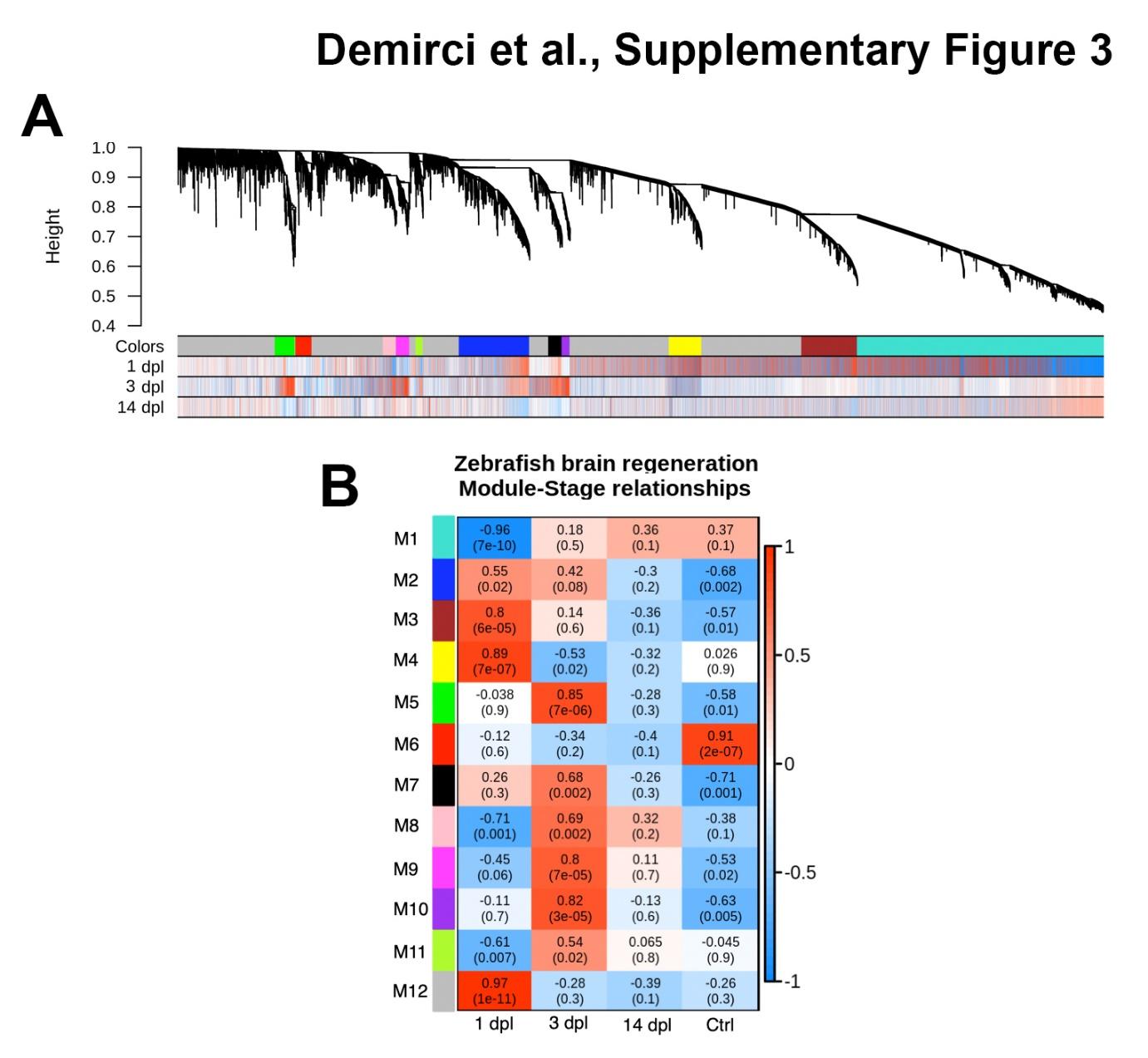
**

**Supplementary Figure 3. Network analysis of zebrafish brain regeneration at three different stages (A)** Hierarchical clustering dendrogram derived from zebrafish gene expression data and showing co-expression modules identified by WGCNA. The first color band under the dendrogram represents the modules. The color bands underneath show the correlation between each gene’s expression and each stage of zebrafish brain regeneration, with a color scale from high positive correlation (red) to high negative correlations (blue). **(B)** Heatmap showing relationships (and corresponding p-values) between modules and stages. The relationship between a module and a stage is defined as the Pearson correlation coefficient between that module’s eigengene score and the dummy binary variable representing the belonging of samples to that stage. The eigengene of a module is defined as the first principal component of the expression matrix projected on that module. Correlation coefficients and associated p-values are calculated using WGCNA. dpl: days post-lesion, M: module, WGCNA: weighted gene co-expression network analysis.

**
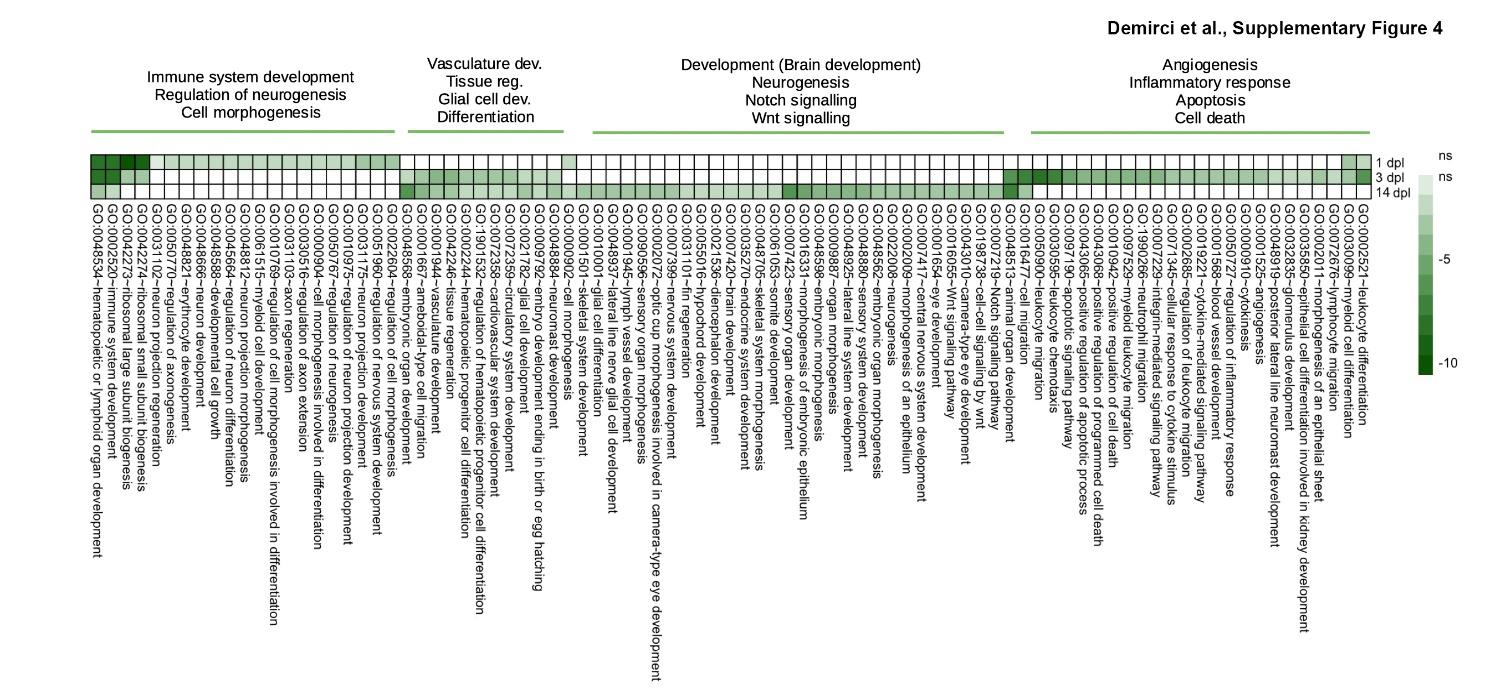
**

### **Supplementary Figure 4. Heatmap representing** **clustering of selected regeneration-related GO-BP terms enriched at three different stages.** dpl: days post-lesion, ns: non-significant.

**
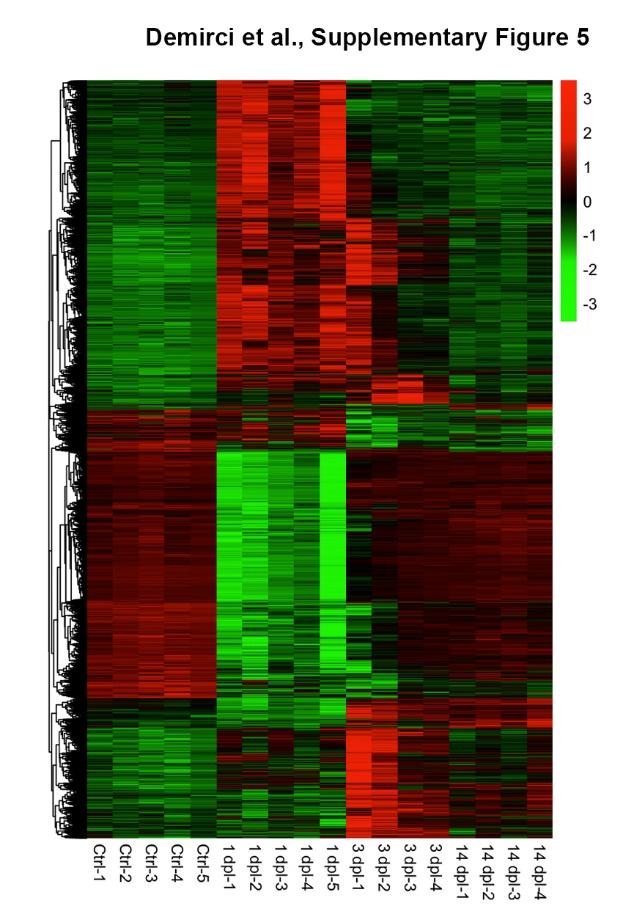
**

**Supplementary Figure 5. Heatmap of all DEGs (9136 genes) identified at three stages of adult zebrafish brain regeneration.** The heatmap shows the z-score of variance-stabilized counts of all DEGs (9136 genes) across three stages. Each column represents a sample, and each row shows a single gene. Red and green shades show high or low relative expressions, respectively. dpl: days post-lesion, Ctrl: control.


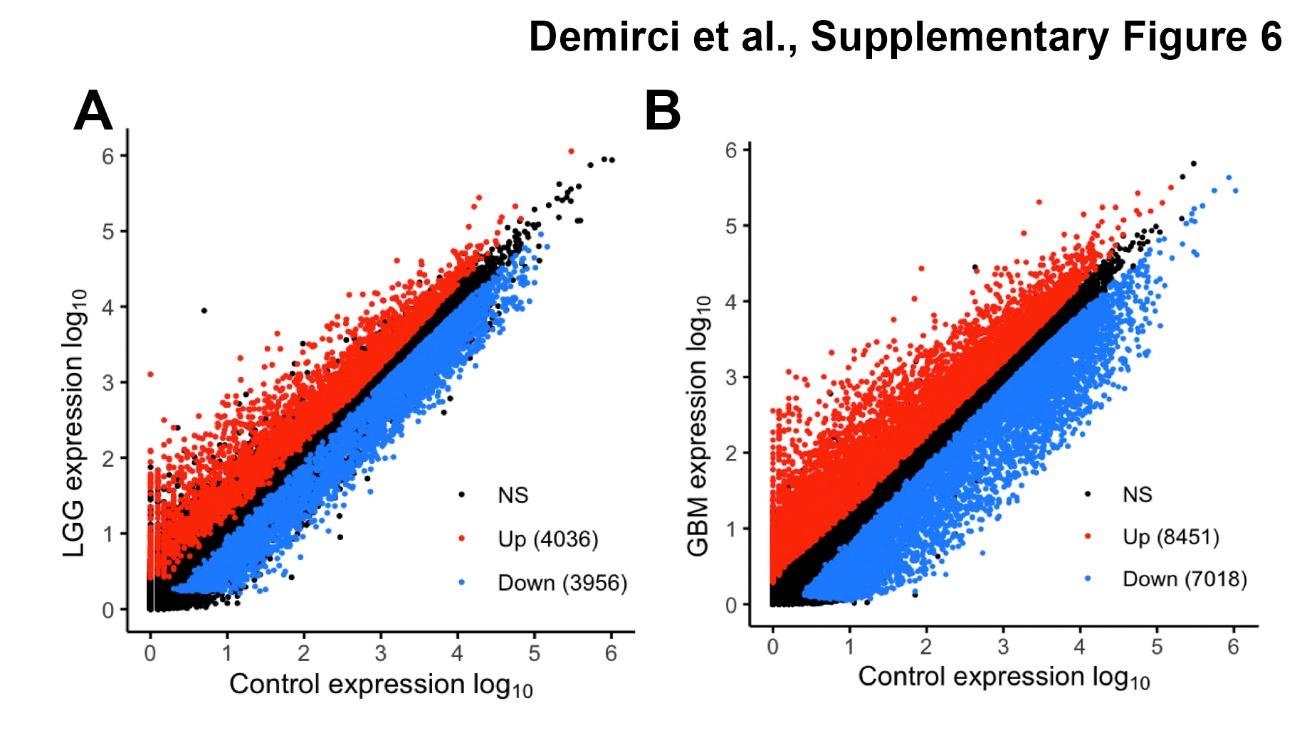


**Supplementary Figure 6.** **Transcriptomic profiling of LGG and GBM.** Scatterplots showing the numbers of upregulated (Up, red) and downregulated (Down, blue) genes in (A) LGG (529 samples) and (B) GBM (165 samples), as compared to normal brain (4 normal brain samples were used for LGG data analysis, 5 normal brain samples were used for GBM data analysis). NS: non-significant, LGG: low-grade glioma, GBM: glioblastoma.


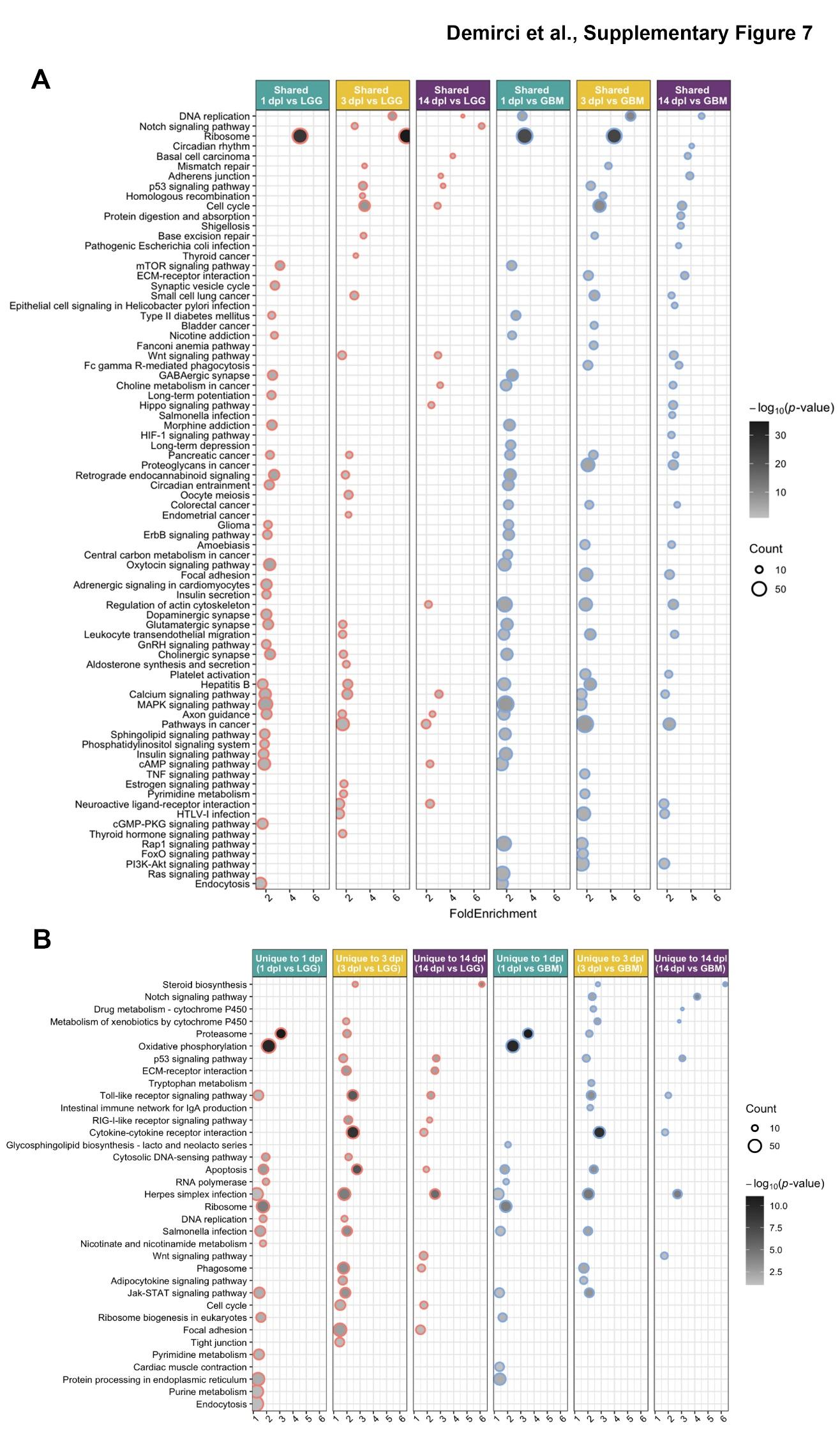


**Supplementary Figure 7.** **KEGG pathway analyses of the comparison of zebrafish brain regeneration with human brain cancers. (A)** DAVID was used to show the 30 most significantly enriched KEGG pathways determined by using the DEGs shared between zebrafish brain regeneration (1 dpl, 3 dpl and 14 dpl) and human brain cancers (LGG and GBM). **(B)** DAVID was used to show all significantly enriched KEGG pathways determined by using the DEGs that are unique to zebrafish brain regeneration and does not exist in LGG or GBM. The heatmap’s scale shows negative log_10_ of EASE p-values for all significantly enriched GO terms. dpl: days post-lesion, LGG: low-grade glioma, GBM: glioblastoma.

**
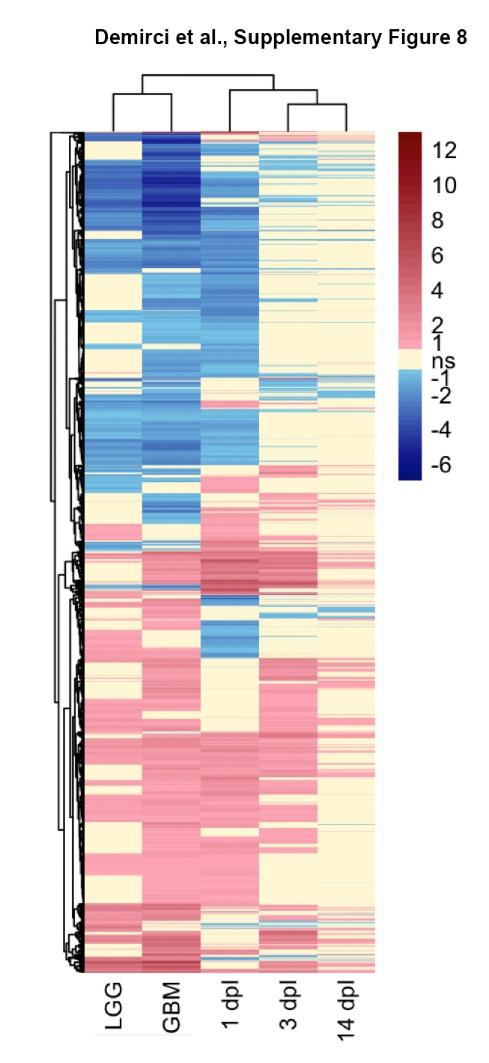
**

**Supplementary Figure 8.** **Heatmap of the DEGs (3615 genes) detected in brain regeneration and brain cancer.** The heatmap shows log_2_ fold changes of 3615 genes that are differentially expressed in at least one stage of adult zebrafish brain regeneration and shared with at least one type of brain cancer. Each column represents a time point of brain regeneration or a cancer type and each row shows a single gene. The log_2_ fold change scale shows red for upregulation, blue for downregulation, yellow for weak regulation (FC < 1.5 in either direction) or statistically non-significant regulation (Benjamini–Hochberg adjusted p-value (FDR) > 0.1). dpl: days post-lesion, LGG: low-grade glioma, GBM: glioblastoma.

### **
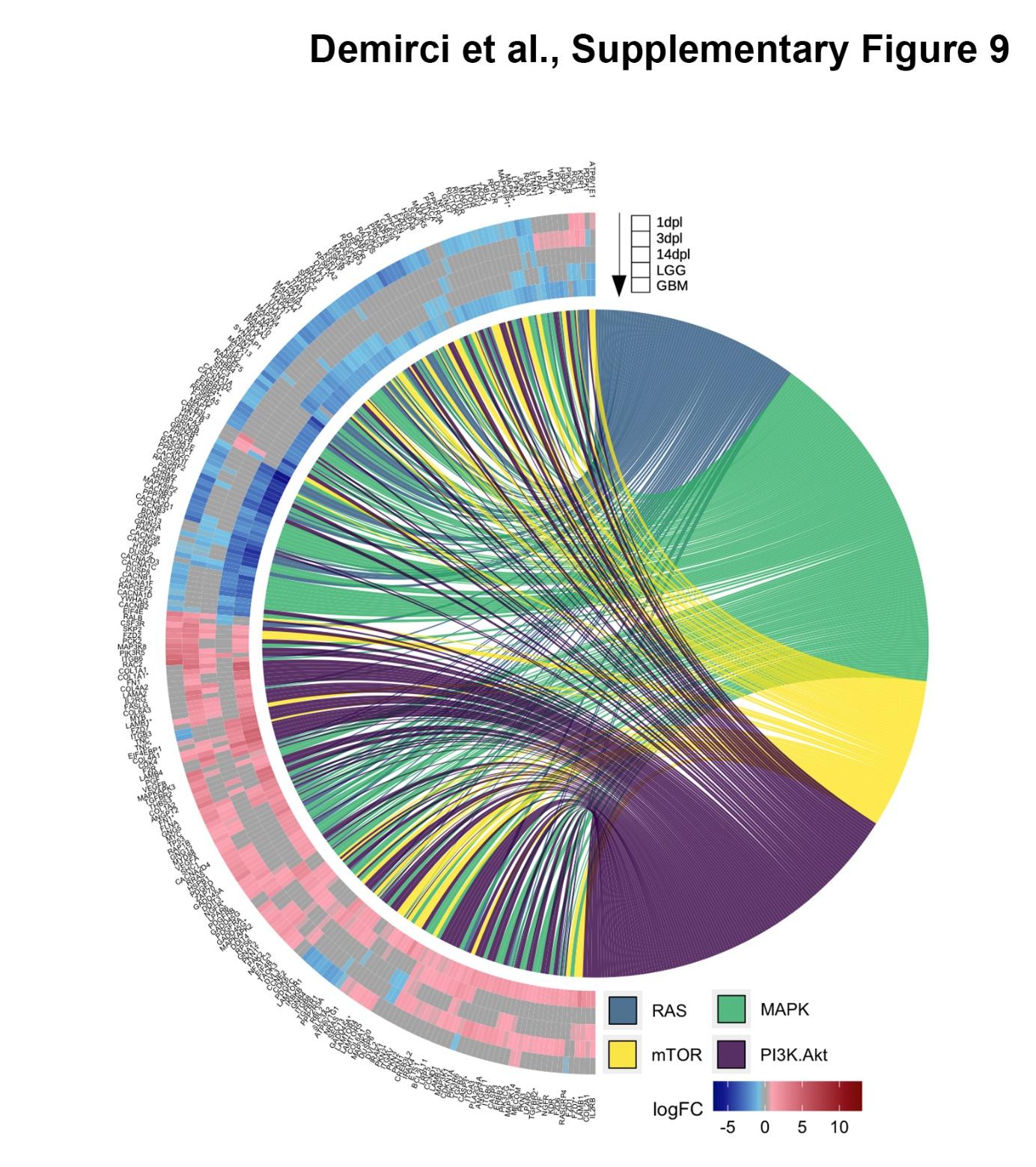
**

### **Supplementary Figure 9. Early wound healing stage of brain regeneration is more similar to LGG and GBM than the proliferation and differentiation stages. This** GOChord plot shows log_2_ fold changes of the genes annotated in selected KEGG pathways RAS, MAPK, mTOR and PI3K-Akt for the three stages of zebrafish brain regeneration and the two types of human brain cancer. Genes are linked to their assigned pathways by ribbons and clustered according to the five log_2_ fold change profiles shown. log_2_ fold changes are shown from the outer to the inner annulus in the following order: 1 dpl, 3 dpl, 14 dpl, LGG and GBM. The log2 fold change scale shows red for upregulation, blue for downregulation, and grey for weak regulation (FC < 1.5 in either direction) or statistically non-significant regulation (FDR > 0.1). An asterisk was appended to human genes associated as orthologs to several zebrafish genes in the list. dpl: days post-lesion, LGG: low-grade glioma, GBM: glioblastoma.
